# Oxidized ATM promotes breast cancer stem cell enrichment through energy metabolism reprogram-mediated acetyl-CoA accumulation

**DOI:** 10.1038/s41419-020-2714-7

**Published:** 2020-07-03

**Authors:** Dan Yang, Meixi Peng, Yixuan Hou, Yilu Qin, Xueying Wan, Pengpeng Zhu, Shuiqing Liu, Liping Yang, Huan Zeng, Ting Jin, Yuxiang Qiu, Qiao Li, Manran Liu

**Affiliations:** 1 Key Laboratory of Laboratory Medical Diagnostics designed by Chinese Ministry of Education, Chongqing Medical University, #1 Yi-Xue-Yuan Rd., Yu-zhong District, Chongqing, 400016 China; 2 Experimental Teaching Center of Basic Medicine Science, Chongqing Medical University, #1 Yi-Xue-Yuan Rd., Yu-zhong District, Chongqing, 400016 China

**Keywords:** Cancer, Cancer metabolism

## Abstract

Cancer stem cell (CSC) is a challenge in the therapy of triple-negative breast cancer (TNBC). Intratumoral hypoxia is a common feature of solid tumor. Hypoxia may contribute to the maintenance of CSC, resulting in a poor efficacy of traditional treatment and recurrence of TNBC cases. However, the underlying molecular mechanism involved in hypoxia-induced CSC stemness maintenance remains unclear. Here, we report that hypoxia stimulated DNA double-strand breaks independent of ATM kinase activation (called oxidized ATM in this paper) play a crucial role in TNBC mammosphere formation and stemness maintenance by governing a specific energy metabolism reprogramming (EMR). Oxidized ATM up-regulates GLUT1, PKM2, and PDHa expressions to enhance the uptake of glucose and production of pyruvate rather than lactate products, which facilitates glycolytic flux to mitochondrial pyruvate and citrate, thus resulting in accumulation of cytoplasmic acetyl-CoA instead of the tricarboxylic acid (TCA) cycle by regulating ATP-citrate lyase (ACLY) activity. Our findings unravel a novel model of TNBC-CSC glucose metabolism and its functional role in maintenance of hypoxic TNBC-CSC stemness. This work may help us to develop new therapeutic strategies for TNBC treatment.

## Introduction

Triple-negative breast cancer (TNBC) is a breast cancer subtype as defined by the lack of estrogen receptor (ER) and progesterone receptor (PR), as well as human epidermal growth factor receptor-2 (HER-2) expressions. TNBC accounts for approximately 15–20% of all diagnosed breast cancer cases, and is more prevalent in younger women (<40 years of age)^1^. TNBC predominantly presents as invasive ductal carcinomas, characterized by poor differentiation, high proliferative capacity, a large overall tumor size^2^, and increased lymph node involvement at the time of diagnosis, contributing to the highest risk of recurrence and metastasis of all breast cancer types at an early time. Previous studies have proposed that in TNBC, cancer stem cells (CSCs) may mediate tumor metastasis, thus contributing to recurrence and chemotherapy resistance^3^. Even more importantly, recent evidence indicates that TNBC-CSCs could be augmented by traditional therapeutic strategies through the hypoxia pathway^4,5^.

Over the years, stem cell metabolism was viewed as an accessory substance of cell fate status rather than a critical regulatory agent. However, an increasing evidence suggests that some intermediates in energy metabolism regulate epigenetic and transcriptional changes associated with stemness maintenance and self-renewal, including *S*-adenosyl methionine (SAM) produced via the one-carbon cycle, acetyl-CoA (Ac-CoA) from glycolysis, α-ketoglutarate (αKG) and flavin adenine dinucleotide (FAD) from the tricarboxylic acid (TCA) cycle, and NAD^+^ from the integration of glycolysis and OXPHOS^6,7^. Histone acetylation is critical in maintaining an open-chromatin structure, and Ac-CoA is a major donor to histone acetylation^8,9^. For example, acetyl-CoA, as the only substrate of acetyl groups for histone and non-histone protein acetylation, in combination with histone acetyltransferases (HATs) and deacetylases (HDACs and sirtuins), could influence the level of histone and non-histone protein acetylation leading to epigenetics regulation. In the mammalian cells cytosol, ATP-citrate lyase (ACLY) cleaves glucose-derived citrate to nuclear acetyl-CoA, which then served as the donor for histone acetylation. Amusingly, acetyl-CoA derived from fatty acid oxidation has no much contribution to histone acetylation^10^. In line with this, human and mouse ESCs^11^, MuSCs^12^, tumor cells^13^, and yeast^14^ possess the similar relationship between glycolysis and histone acetylation. Acetate, a precursor of acetyl-CoA, can stimulate histone acetylation and delay differentiation and loss of pluripotency^11^. However, the roles of metabolites in TNBC-CSCs are largely unknown.

Hypoxia, as a widespread stimulator, is related to many physiological and pathological processes, such as cancer, stroke, hypertension, and diabetes. Intratumoral hypoxia is a common feature in solid cancers, which significantly facilitates an aggressive tumor phenotype and therapeutic resistance, resulting in poor patient survival^15,16^. Low oxygen tensions can straight away affect the pluripotency or differentiation of stem cells. For instance, hypoxic culture prevents differentiation of human embryonic stem (hES) cells^17^ and promotes an undifferentiated and multipotent status in mesenchymal stem cells (MSC)^18^. Hypoxia was found to promote enrichment of CSCs through the HIF1α/2α and AKT/mTOR/β-catenin pathway^19,20^. Thus, hypoxia has become a hot topic of clinically targeted therapy^21,22^.

ATM is originally identified as the product of the gene mutated in ataxia telangiectasia (A–T), manifested by progressive neuronal degeneration, cancer predisposition, immunodeficiency, and hypersensitivity to radiotherapy^23^. ATM has previously been considered to be located in the nucleus to repair DNA double-strand breaks (DSBs) after being activated by DNA damage^24^. Recently, it has been reported that there is cytoplasmic ATM under hypoxia^25^, called DNA damage-independent ATM (or DSB-independent ATM, oxidized ATM), and involves in various processes and responses, some of which may contribute to intracellular redox homeostasis and metabolism reprogramming^25,26^. Some studies show that oxidized ATM plays a key role in self-renewal capacity of embryonic stem cells^27^, and involves in regulating tumor malignant phenotypes^25^. However, the functions of oxidized ATM in TNBC-CSCs under hypoxia are unclear.

Here, we investigated whether hypoxia contributes to activation of oxidized ATM in TNBC-CSCs and revealed that oxidized ATM-mediated energy metabolism reprogramming (EMR) plays a crucial role for maintenance of TNBC-CSC stemness under hypoxia. We found that oxidized ATM induces a specific EMR, which possesses a high-level glycolysis and a relative high activated mtOXPHOS in TNBC-CSCs, which facilitates glycolytic flux to pyruvate and citrate and then fuels acetyl-CoA production in the cytoplasm. The accumulation of cytoplasmic acetyl-CoA results in histone H4K8ac, H4K12ac and H4K16ac acetylation, and a high level of CSC-associated gene expressions. Therefore, our study demonstrates that metabolic reprogramming mediated by oxidized ATM activation plays a major role in maintenance of TNBC-CSC stemness.

## Materials and methods

### Cell culture, chemical inhibitors, shRNAs, and plasmids

Human breast cancer cells Hs578T and BT549 were obtained from ATCC (American Type Culture Collection). Hs578T and BT549 cells were routinely cultured in RPMI-1640 medium (Gibco) with 10% fetal bovine serum (FBS, Gibco) in a humidified atmosphere of 5% CO_2_ at 37 °C with 21% O_2_ or 1% O_2_. KU60019, BMS303141, Etomoxir (ETO), Metformine, 2-deoxy-d-glucose (2-DG), and acetate was purchased from Sigma; UK5099 was the product of Selleck Corp. STAT5-IN-1 was a product of BioVision. The lentivirus expression vector of shRNA against ATM, GLUT1, PKM2, PDHa, and FOXP3 was obtained from GenePharma (Shanghai, China). The core sequences of shRNA against ATM, GLUT1, PKM2, PDHa, FOXP3, and control shRNA sequences are listed in Table 1. To acquire the luciferase reporter of GLUT1, PKM2, and PDHa, the target promoter fragment (−1200 to +100) was amplified by PCR and inserted into the pGL3-basic reporter plasmid.

**Table 1:** Core sequences of shRNA against target genes.

Target gene	shRNA sequences
NC	5′-TTCTCCGAACGTGTCACGT-3′
	5′-GCTGTTACCTGTTTGAAAA-3′
ATM	5′-GCCGTCAACTAGAACATGATA-3′
	5′-CCAAGGTCTATGATATGCTTA-3′
	1# 5′-GTTCGGAGGTTTGATGAAATC-3′
PKM2	2# 5′-CTACCACTTGCAATTATTTGA-3′
	3# 5′-CCACTTGCAATTATTTGAGGA-3′
	1# 5′-GATGGGCTGGCGGTAGGCGG-3′
GLUT1	2# 5′-GCATGTGCTTCCAGTATGT-3′
	3# 5′-GAATTCAATGCTGATGATGA-3′
	1# 5′-CTGGTAGCATCCCGTAATTT-3′
PDHa	2# 5′-CCAATCAGTGGATCAAGTTT-3′
	3# 5′-GAATGGAGTTGAAAGCAGAT-3′
	1# 5′-TGTTTCATGAGAACAAAGCACTTGTGCAGA-3′
FOXP3	2# 5′-GCTTTCATGAGAAGCTGGTGCATGAAATGT-3′
	3# 5′-GAATTCATGAGAAAGACTTCTTCCGTTTGT-3′
	1# 5′-GCATGTCGCAAGGCTGTGT-3′
USP13	2# 5′-CGATTTAAATAGCGACGATTA-3′
	3# 5′-GCCAGTATCTAAATATGCCAA-3′

### Mammosphere formation assay

Breast cancer cells Hs578T and BT549 were dissociated into single cells by 0.05% trypsin-EDTA solution and plated into six-well plates coated with 2% poly-HEMA (Sigma) at a density of 1 × 10^4^ cells/mL in primary culture and 5 × 10^3^ cells/mL in following passages in hypoxia (1% O_2_). The incubation of mammospheres was carried out under hypoxia (1% O_2_).

For the mammosphere culture of breast tumor cells from clinical patients, the tumor tissues were minced and digested with 1 mg/mL of type 1 collagenase (Sigma) at 37 °C for 30 min to prepare single-cell suspensions. Mammosphere cells were grown in Complete Mammm Cult Medium (Stem Cell Technologies) or a serum-free Dulbecco's Modified Eagle's medium (DMEM)/F12 medium supplemented with B27 (Invitrogen), 20 ng/mL EGF, 20 ng/mL bFGF, 0.4% BSA, 2 μg/mL heparin, and 5 μg/mL insulin. The incubation of mammospheres was carried out under hypoxia (1% O_2_). Mammospheres were passaged every 4 days. The numbers of secondary-generation spheres (at least 50 μm diameter) were counted using an OLYMPUS IX70 microscope (Tokyo, Japan) fitted with graticule at ×100 magnification. The percentage of mammosphere forming efficiency (MFE) was calculated as previously described^28^. The average size of the randomly selected mammospheres (*N* = 30) was calculated. The percentage of MFE (%) from primary to tertiary generation indicated the self-renewal capacity of each kind of mammospheres. Mammosphere cell growth was determined by cell numbers contained within each generation of mammospheres. All experiments were performed at least three times.

### Flow cytometry analysis

For CSCs, the secondary-generation spheres were trypsinized into single cells, washed using Hanks balanced salt solution with 2% FBS (HF solution), and stained freshly with antibodies against CD24 (BD Biosciences, USA) and CD44 (BD Biosciences, USA) according to the manufacturer’s manuals. The CD44^+^/CD24^−/lower^ cells were determined by flow cytometry analysis.

Cell proliferation and cell death were also assessed via flow cytometry analysis. Ki67 (1:100, Abcam, Cat#ab245113) was used to evaluate the cell proliferation ability. Cell death was measured by double staining the cells with Annexin-PE/7-AAD (Keygen, Nanjing, China) using a FACS Calibur flow cytometer (BD). All experiments were performed at least three times.

### Immunohistochemistry (IHC) and immunofluorescence (IF)

The paraffin-embedded tumor specimens were sectioned at 4 μm thickness and heated for antigen retrieval at 95 °C in citric acid buffer (pH 6.0). The immunohistochemical was operated as described previously^29^. After treating with 3% H_2_O_2_, the sections were blocked with 5% goat normal serum, and incubated with primary antibody against GLUT1 (1:100, Proteintech, Cat#21829-1-AP), PKM2 (1:100, Abcam, Cat#ab137852), PDHa (1:200, Abcam, Cat#ab168379), H4ac (1:200, Millipore, Cat#PA5-40083), SOX2 (1:100, Abcam, Cat#ab93689), KIF4 (1:100, Abcam, Cat#ab124903), OCT4 (1:100, Abcam, Cat#ab18976), and c-MYC (1:100, Abcam, Cat#ab32072) separately. Image-Pro plus 6.0 software was employed to quantify the IHC staining. IHC staining intensities (I) were scored as 0, 1+, 2+, 3+, and 4+. The percentage of the stained area (A) was scored as 1 (0–25%), 2 (26–50%), 3 (51–75%), and 4 (76–100%). The sum of the intensity and percentage scores (I+A) was used as the final IHC score, which was defined as follows: 1–2, negative; 3–4, weak; 5–6, moderate; 7–8, strong. All experiments were performed at least three times.

For IF staining, the secondary-generation spheres were collected and resuspended with phosphate-buffered saline (PBS). Mammospheres were placed on coverslips coated with 0.1% poly-l-lysine and then fixed within 4% paraformaldehyde for 20 min at room temperature. After washing with PBS, cells were treated with 0.1% Triton-100 for 15 min at room temperature and incubated with 5% goat serum solution for 30 min at 37 °C. Cells were stained with primary antibodies against H4ac (1:200) at 4 °C overnight, followed by a Cy3-labeled goat anti-rabbit secondary antibody (1:200, Sigma) for 1 h at 37 °C in a humidified incubator. Sections were mounted in aqueous medium containing DAPI as a nuclear counter stain (Vector LABS). A negative control was used to ensure the specificity of fluorescent immunostaining by replacing the primary antibody with a normal rabbit IgG. A Nikon Eclipse 80i microscope (Eclipse 80i, Tokyo, Japan) was used to take immunofluorescent images. All experiments were performed at least three times.

### Western blot analysis

The secondary-generation mammospheres were lysed with cold RIPA buffer (Boster, China), and cell lysates were separated by SDS-PAGE. Nuclear extracts from the cortex were obtained using nuclear and cytoplasmic protein extraction Kit (Beyotime, Shanghai, China). Equal amounts of proteins were loaded into SDS-PAGE (8, 10, or 12%), electrophoresed, and transferred onto PVDF membranes (Millipore, Temecula, CA). Proteins were incubated with specifically primary antibodies, and β-actin and H4 were used as a loading control. The primary antibodies used in this analysis are as follows: ATM (1:1000, Abcam, Cat#ab32420), p-ATM (1:1000, Abcam, Cat#ab81292), c-MYC (1:1000, Abcam, Cat#ab32072), OCT4 (1:1000, Abcam, Cat#ab18976), KLF4 (1:1000, Abcam, Cat#ab124903), SOX2 (1:1000, Abcam, Cat#ab93689), HDAC1 (1:1000, Abcam, Cat#ab213701), HDAC2 (1:1000, Abcam, Cat#ab32117), HK2 (1:1000, Abcam, Cat#ab209847), ENO1 (1:1000, Abcam, Cat#ab155102), PKM2 (1:1000, Abcam, Cat#ab137852), LDHA (1:1000, Abcam, Cat#ab101562), PDHa (1:1000, Abcam, Cat#ab168379), γH2AX (1:1000, Abcam, Cat#ab81299), H2AX (1:1000, Abcam, Cat#ab20669), H4K16ac (1:1000, Abcam, Cat#ab109463), H4K12ac (1:1000, Abcam, Cat#ab177793), H4K8ac (1:1000, Abcam, Cat#ab45166), β-Actin (1:1000, CST, Cat#3700), PFKP (1:1000, CST, Cat#12746), CS (1:2000, CST, Cat#14309), ACLY (1:2000, CST, Cat#4331), H4ac (1:10000, Millipore, Cat#PA5-40083), GLUT1 (1:1000, Proteintech, Cat#21829-1-AP), KLF4 (1:1000, Abcam, Cat#ab124903), FOXP3 (1:1000, Abcam, Cat#ab20034), STAT5 (1:1000, Abcam, Cat#ab227687), p-STAT5 (1:1000, Abcam, Cat#ab32364), 53BP1 (1:2000, Abcam, Cat#ab175933), and H4 (1:200, Beyotime, Cat#AH436).

### qRT-PCR assay

Total RNA was isolated from secondary-generation mammospheres or breast tumor tissues using Trizol (Invitrogen). RNA was subjected to reverse transcription reactions by using the Prime Script RT reagent Kit (Takara Bio, Dalian, China). qRT-PCR was performed by CFXConnect™ real-time PCR detection system (Bio-Rad, Hercules, CA, USA) using SYBR® Premix Ex Taq™ II (Takara Bio). The primers used in qRT-PCR are listed in Table 2. β-Actin was used as a loading control. All experiments were repeated at least three times.

**Table 2:** Primer sequences used for qRT-PCR analysis.

Gene	Primer sequence
OCT4	F: TGGGCTCGAGAAGGATGTG
	R: GCATAGTCGCTGCTTGATCG
KLF4	F: GAACTGACCAGGCACTACCG
	R: TTCTGGCAGTGTGGGTCATA
c-MYC	F: AAAGGCCCCCAAGGTAGTTA
	R: GCACAAGAGTTCCGTAGCTG
SOX2	F: GCACATGAACGGCTGGAGCAACG
	R: TGCTGCGAGTAGGACATGCTGTA
β-Actin	F: CTGGAAGGTGGACAGCGAGG
	R: CTGGAAGGTGGACAGCGAGG
SIRT1	F: GATGACGATGACAGAACGTCACA
	R: GGATCGGTGCCAATCATGAG
HK2	F: TGCTTGCCTACTTCTTCACG
	R: CATCTGGAGTGGACCTCACA
PKM2	F: AAATCACGCTGGATAACGCCTA
	R: AGCCACAGGATGTTCTCGTCA
LDHA	F: ATCAAACTCAAAGGCTACACA
	R: TACTCTCTGCCAAATCTGCTAC
ENO1	F: CAATGTCATCAATGGCGGTTC
	R: GTAAACCTCTGCTCCAATGCG
PDHA	F: ACCCCACAGACCATCTCATCACA
	R: AAAGTAAAGCCGTGAGCCCGGTA
PDHB	F: GTATGGATGAGGAGCTGGAAAG
	R: GCCCTCGACTAACCTTGTATG
PDK	F: ATGAAAGAGATC AACCTGCTTCC
	R: GGCTCTGGACATACCAGC TC
CS	F: TGGCTAACACAGCTGCAGAA
	R: CATAGCCTGGAACAACCCGT
PFKP	F: CGGAAGTTCCTGGAGCACCTCTC
	R: AAGTACACCTTGGCCCCCACGTA
ACLY	F: CATATCCAGAGGAAGCCTACATTG
	R: ATGGTCCAGATCCTCCCTTT

### Luciferase reporter assay and chromatin immunoprecipitation (ChIP) assay

Cells were plated at a density of 1 × 10^5^ cells in a 24-well plate. Transfection were performed by Lipofectamine 3000 (Invitrogen, USA) using 1 μg of the reporter, 50–200 ng of expression plasmids or control vector. Luciferase activity was normalized for transfection by using β-galactosidase reporters as an internal control. The mutated sequences used in luciferase reporter assay were shown as the following: Mut of the E1 of GLUT1: 5′-acgggcg-3′; Mut of the E1 of PKM2: 5′-gcggaag-3′; Mut of the E1 of PDHa: 5′-aggggtg-3′; and Mut of the E2 of PDHa: 5′-acggttg-3′.

ChIP assay was performed using a Thermo Fisher Biotechnology kit as described elsewhere. Chromatin prepared from cells in a 30 cm^2^ bottle was used to determine total DNA input and for overnight incubation with the specific antibody or with normal rabbit IgG. The specific primers used to amplify the fragment of FOXP3-binding motif in the human GLUT1, PKM2, and PDHa promoter in PCR are listed in Table 3. All experiments were performed at least three times.

**Table 3:** Primer sequences used for PCR analysis in ChIP assay.

Gene	Primer sequences
GLUT1-E1	F: 5′-GAGGACTACAAGGCGGATGGC-3′
	R: 5′-TTTGGGAAATGGCGGTGC-3′
PKM2-E1	F: 5′-ATGTCACCGAGCAAAGAA-3′
	R: 5′-ACCACCTGAGAAAGCCAC-3′
PDHa-E1	F: 5′-CCCTCTTGCTGCTGTCTACACC-3′
	R: 5′-ACCTCAACTCCCTGCGGCTCA-3′
PDHa-E2	F: 5′-GCTCCACAGACCACCGCTCCCT-3′
	R: 5′-CGCTCCCCGCCAGCTTTGTGAC-3′

### Metabonomics analysis

Liquid chromatography with tandem mass spectrometry metabolomics were performed by Novogene (Beijing, China). After harvesting 1 × 10^6^ CSCs, a series of cell processing were followed, such as annihilation with quenching buffer (60% (v/v) methanol plus 0.85% (wt/v) sodium bicarbonate) at −40 °C, washing with PBS, resuspension of cells with methanol at −80 °C, and then use of cells for metabolomics assay. All experiments were performed at least three times.

### Detection of glucose consumption, ATP level, and intermediate productions

The levels of glucose, lactate, and pyruvate were separately determined using the Glucose Assay Kit, Lactate Assay Kit, and Pyruvate Assay Kit (Jiancheng Bioengineering Institute, Nanjing, China). The levels of succinate and fumarate were separately measured by using the Succinate Colorimetric Assay Kit and the Fumarate Assay Kit (Sigma, St. Louis, MO). The levels of acetyl-CoA were tested using the Acetyl-Coenzyme assay kit and citrate levels were tested using the Citric Acid Assay Kit (Solarbio, China). The ATP products were measured using the ATP Assay Kit, and the mitochondrial membrane potential was determined using the JC-1 Dye (Beyotime, China). All these measurements follow their respective manufacturer’s protocols and were normalized by cell numbers or the quality of clinical sample. All experiments were performed at least three times.

### Seahorse metabolic flux analyzer

The extracellular acidification rate (ECAR) and oxygen-consumption rate (OCR) of breast CSC cultured under the indicated conditions were determined using a Seahorse XF24 Extracellular Flux Analyzer (Seahorese Bioscience). In brief, breast CSCs were plated in wells coated with 2% poly-HEMA (Sigma) in full growth medium at a concentration of 1 × 10^4^ cells per well. During the analysis of CSC, 0.4% BSA was added to the XF24 medium. Measurements of OCR and ECAR were made after the addition of the inhibitors oligomycin (2 μg/mL, Sigma), carbonyl cyanide 4-(trifluoromethoxy) phenylhydrazone (FCCP; 2 μM, Sigma), rotenone (100 nM, Sigma) combined with antimycin A (2 μM, Sigma), and 2-deoxyglucose (2-DG, 20 mM). The progress curve of OCR is annotated to show basal respiration, ATP production, maximal respiration after addition of FCCP, and spare respiratory capacity after addition of rotenone and antimycin A. The progress curve of ECAR is annotated to show glycolysis, glycolytic capacity after the addition of oligomycin, and glycolytic reserve. ECAR and OCR measurements were normalized to those of 1 × 10^6^ CSC. All experiments were performed at least three times.

### Stable isotope tracer analysis

For the analysis of TCA-cycle intermediates, CSCs were cultured and labeled in the standard serum-free DMEM/F12 media containing 17.5 mM ^13^C_6_-glucose (Cambridge Isotope Laboratories). For time course experiments, the secondary-generation mammospheres were cultured in serum-free DMEM/F12 medium for 2 days. The BCSCs were washed three times in medium without glucose, and then the cells were cultured for another 8 h in medium containing 17.5 mM ^13^C_6_-glucose. At the time of collection, the cells were washed three times with ice-cold PBS and extracted with 80% methanol on ice. Extracts were dried by centrifugation and stored at −80 °C until further processing. The intermediates were normalized according to internal standard solution containing [U-13C] fumarate, succinate-d4, and citrate-d4 at 1 mM each. For the analysis of lactate through glycolysis, BCSCs were cultured in the medium containing 1,2-^13^C_2-_glucose (Cambridge Isotope Laboratories). At the time of collection, the culture medium was centrifuged, transferred to a new Eppendorf tube, and stored at −80 °C until further processing.

### Xenograft models

Animal experiments were performed in compliance with guidelines on animal care and use established by the Chongqing Medical University Experimental Animal Management Committee. The BT549 mammosphere cells were injected subcutaneously into the groin of 4-week-old athymic nude mice (1 × 10^5^ cells per mouse). The longest diameter and its widest vertical width of tumor were measured 3 days each with a dialcaliper. Tumor volume was calculated using the equation *L* × *W*^2^/2. When tumor volume reaches around 50 mm^3^, mice were randomized into five groups and administered with KU60019 (50 mg/kg, three times per week), UK5099 (10 μg/kg, once every 2 days), acetate (250 mg/kg, three times per week) alone or in combination, via intraperitoneal (i.p.) injection. Four weeks after continuous administration, mice were euthanized, and tumors were surgically isolated and weighted. A suitable amount of xenograft tumor tissues was taken for the determination of acetyl-CoA and the residual tissues were fixed in 4% paraformaldehyde and subjected to immunohistochemistry to detect GLUT1, HK2, SIRT1, SOX2, and H4ac levels.

### Statistical analysis

Statistical analysis was done using SPSS standard version 17.0 software and measurements are presented as means ± SD. For comparisons between multiple groups, ANOVA followed by the Student–Newman–Keuls multiple comparisons test was used; and for single comparisons between two groups, Student's *t-*test was used. A value of *P* < 0.05 was considered to be significant.

## Results

### Hypoxia facilitates enrichment of CSC derived from triple-negative cancer cells (TNBC-CSCs)

**Fig. 1:**
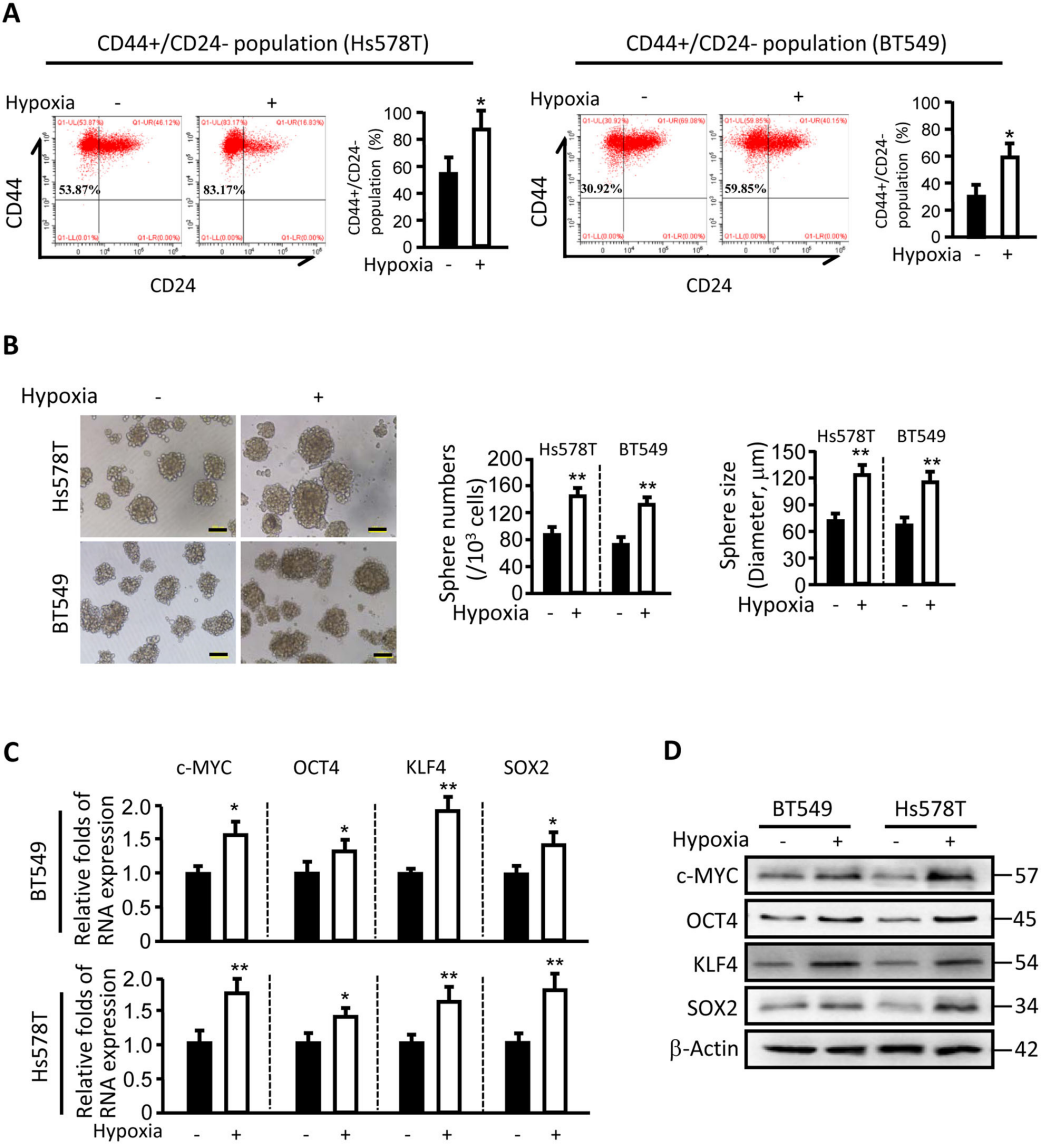
Enrichment of TNBC-CSC in hypoxia conditions. **a** Hs578T and BT549 cells were exposed to 21 or 1% O_2_ for 24 h; CD44^+^/CD24^−^ cells in both Hs578T and BT549 cell populations were detected by flow cytometry (*P < 0.05). **b** Representative images of mammospheres formed from Hs578T and BT549 cells in 1% O_2_ condition (scale bar, 100 µm). The quantification of mammosphere formation efficiencies (sphere number) and the mean sphere sizes was shown (**P < 0.01). **c**, **d** qRT-PCR (**c**) and western blotting (**d**) were employed to determine the expressions of the indicated CSC-associated genes in mammospheres of Hs578T and BT549 cells under hypoxia conditions (**P < 0.01).

Intratumoral hypoxia is a common feature in solid cancers. It has been demonstrated that hypoxia in breast tumors is linked to poor patient survival rate and recurrence, and CSCs may play a crucial role in tumor recurrence^15^. To figure out whether hypoxia increases breast CSCs, which are CD44^+^/CD24^−^, we detected CSC populations in triple-negative cancer cells (Hs578T and BT549) after hypoxia treatment. As shown in Fig. 1a, when Hs578T and BT549 cells were exposed to 1% O_2_, the percentage of CD44^+^/CD24^−^ cells was markedly increased. Similarly, the mammosphere formation and sizes were also significantly increased under hypoxic conditions in both Hs578T and BT549 cells (Fig. 1b). Accordingly, the enhanced mRNA and protein expression levels of stem cell-associated genes, including c-Myc, Oct4, Klf4, and Sox2, were detected (Fig. 1c, d). These data indicate that hypoxia promotes enrichment and stemness of TNBC-CSC.

### Oxidized ATM is required for TNBC-CSC enrichment

**Fig. 2:**
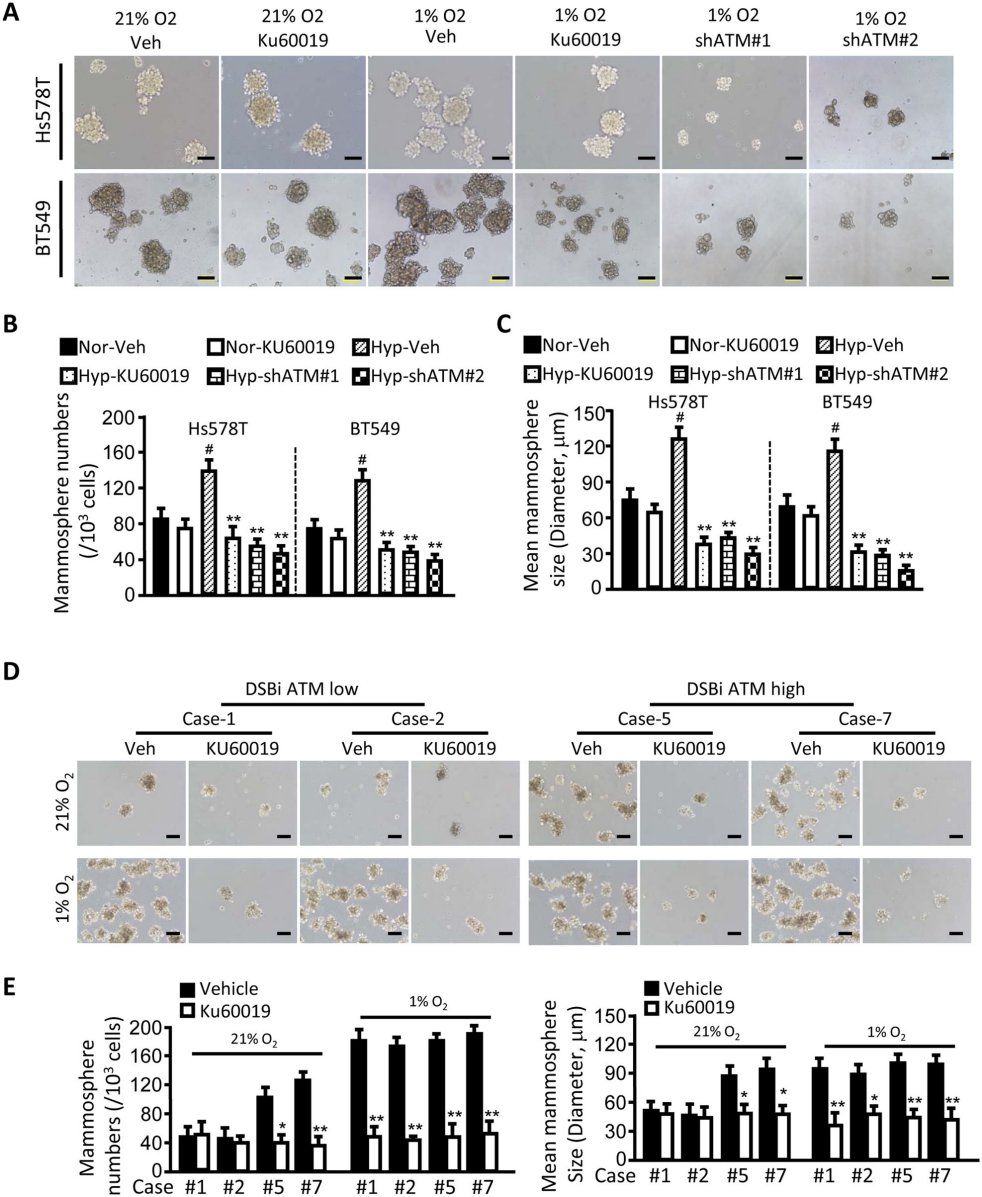
Oxidized ATM promotes TNBC-CSC enrichment. Hs578T and BT549 cells, and ATM-silenced Hs578T and BT549 cells by transfectting two different shRNAs against ATM (shATM#1 and shATM#2) were suspensded in culture in CSC medium under normaxia (21% O_2_) or hypoxia (1% O_2_) and treated with or without ATM inhibitor KU60019 (ATM inhibitor, 10 µM). **a** The representative images of mammospheres are shown (scale bar, 100 µm). **b** The quantification of mammosphere formation efficiency. Data are shown as the mammospheres number/1000 seeded cells ± SEM from three independent experiments (**P < 0.01) (Nor normoxia, Hyp hypoxia). **c** The quantification of mammosphere sizes. The data represent means ± SD from three independent experiments (*P < 0.01, hypoxia vs normoxia; **P < 0.01, vs hypoxic control). **d** The representative images of mammospheres formed from breast tumor with low oxidized ATM and high oxidized ATM (scale bar, 100 µm) under normoxia or hypoxia condition and treatment with or without KU60019 (10 μM). **e** The mammosphere sphere number and mean sphere sizes of **d** were quantified (*P < 0.05, **P < 0.01).

ATM could be activated under hypoxia, and ATM knockdown may result in a strong impairment of tumor malignant phenotypes^25^. To understand whether oxidized ATM contributes to TNBC-CSC stemness maintenance, we evaluated the potential effects of oxidized ATM on CSC. First, we observed that a weak oxidized ATM (p-ATM (s1981), a hallmark of ATM activation by oxidative stress) existed in TNBC-CSC in normoxic CSCs, and hypoxia significantly enhanced p-ATM in CSCs with no DNA damage detected by γH2AX and 53BP1 (the known biomarker of DSBs) (Supplementary Fig. S1A), suggesting that hypoxia stimulated the activation of oxidized ATM kinase in TNBC mammospheres. Thus, it raised a hypothesis that hypoxia-mediated activation of oxidized ATM may involve in the maintenance of TNBC-CSC stemness. As expected, functional inhibition of oxidized ATM using ATM inhibitor KU60019 or silencing endogenous ATM (Supplementary Fig. S1B) caused a marked drop in mammosphere formation of Hs578T and BT549 cells in hypoxia rather than in normoxia (Fig. 2a–c). Correspondingly, the expressions of CSC-associated factors (e.g. c-Myc, Oct4, Klf4, and Sox2) in Hs578T and BT549-derived mammospheres were also reduced (Supplementary Fig. S1C). To expand these findings, a relationship between oxidized ATM expression and CSC enrichment was investigated using breast tumor tissues from patients. The breast tumor cells with a higher level of oxidized ATM (e.g. cases 5 and 7) (Supplementary Fig. S1D) had a stronger CSC sphere formation potential than the case with a lower level of oxidized ATM (e.g. cases 1 and 2) (Supplementary Fig. S1D), and KU60019 tended to impede the CSC sphere formation derived from oxidized ATM higher tumor than that lower tumor in normoxia (Fig. 2d, e). Hypoxia could obviously stimulate the CSC sphere formation of oxidized ATM lower tumor, but it only had mild induction effects on a high level of oxidized ATM-tumors (Fig. 2d, e), which are mainly due to hypoxia treatment efficiently stimulating the expression of oxidized ATM in oxidized ATM low group than that in oxidized ATM high group. Also, KU60019 treatment largely decreased BCSC mammospheres for both oxidized ATM higher and oxidized ATM lower tumors in hypoxia (Fig. 2d, e). Thus, these data indicate that the oxidized ATM is required for TNBC-CSC enrichment.

### Oxidized ATM induces EMR

**Fig. 3:**
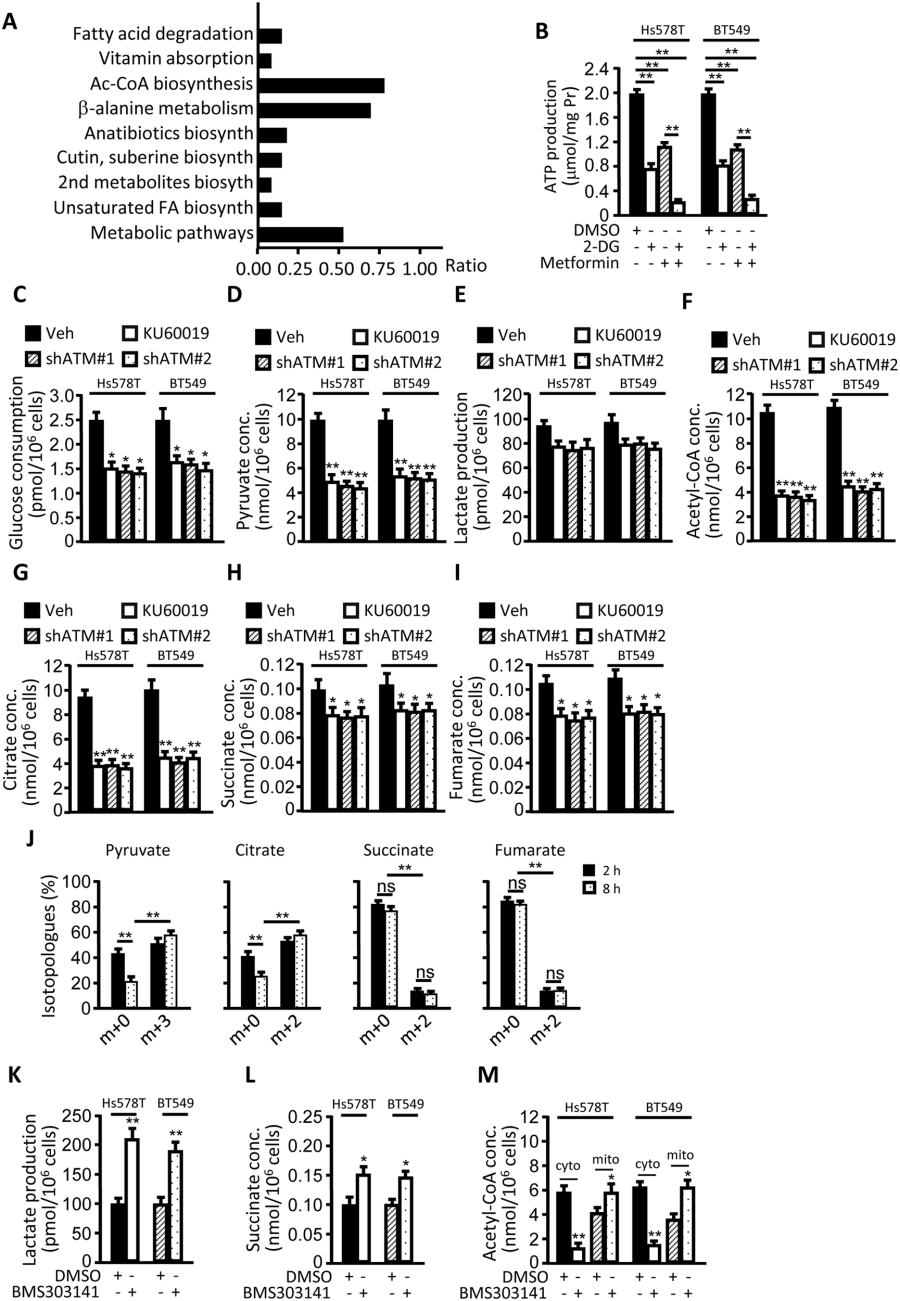
Oxidized ATM induces EMR of CSC. **a** Metabonomics analysis was performed to identify the changed metabolites between oxidized ATM-activated CSC and -inactivated CSC derived from Hs578T under hypoxia, and relative metabolic signaling pathways were enriched by KEGG analysis. Ratio represented the number of changed metabolites to the total number of metabolites in the pathway. **b**–**i** Hs578T and BT549 cells, and ATM-silenced Hs578T and BT549 cells were suspended in culture in CSC medium under hypoxia and treated with or without indicated reagents (e.g., 2-deoxyglucose (2-DG, 5 mM), metformin (3 mM), and/or KU60019 (10 µM)). The relative of ATP products (**b**) and metabolic index were checked. Glucose consumption of mammospheres (**c**); the productions of pyruvate (**d**), lactate (**e**), acetyl-CoA (**f**), citrate (**g**), succinate (**h**), and fumarate (**i**) were shown. **j** CSC incorporation of ^13^C_6_-labeled glucose was added into CSC medium and pulsed for up to 8 h; the isotopolog distribution for pyruvate, citrate, succinate, and fumarate with two ^13^C atoms in sphere cells was shown. **k**–**m** CSCs were treated with or without BMS303141 (an inhibitor of ATP-citrate lyase, 20 μM); lactate, succinate, cytosolic, and mitochondrial acetyl-CoA concentrations were determined (*P < 0.05, **P < 0.01).

The survival of chemotherapy-resistant breast CSCs may rely on glycolysis^30^. To understand the metabolic manner of hypoxic TNBC-CSCs, we analyzed the metabolic characteristics of oxidized ATM-activated CSC and oxidized ATM-inactivated CSC derived from Hs578T using metabonomics. There was activated acetyl-CoA biosynthesis in oxidized ATM-activated CSC (Fig. 3a). Acetyl-CoA was synthesized through TCA cycle; we asked whether mtOXPHOS is the main metabolic pattern in BCSC.

Thus, we checked the ATP levels in CSCs derived from oxidized ATM activated or inactivated, and endogenous ATM knocked down breast cancer cells. In mammosphere cells derived from Hs578T and BT549, the productions of cellular ATP were significantly decreased by 2-deoxy-d-glucose (2-DG, a glucose analog that inhibits glycolysis), and mildly reduced under metformin (an inhibitor of mtOXPHOS) treatment; the ATP levels were further decreased under joint-treatment of 2-DG and metformin (Fig. 3b), implying that hypoxic TNBC-CSCs mainly rely on glycolysis and mtOXPHOS is partially activated in TNBC-CSC. Likewise, basal O_2_ consumption (OCR, an indicator for oxidative phosphorylation (mtOXPHOS)) had no significant change (Supplementary Fig. S2A), the basal media acidification (ECAR, an indicator of glycolysis) and OCR/ERAR ratio in CSCs tended to decrease with loss of oxidized ATM activation or silencing of endogenous ATM in CSC (Supplementary Fig. S2B, C). These data indicate that glycolysis is the main metabolic mode in oxidized ATM-activated BCSCs.

However, it raised another interesting question that why the synthesis of acetyl-CoA was increased in hypoxic TNBC-CSCs, and what was the role of increased acetyl-CoA for TNBC-CSCs. Next, we detected the metabolites concerning glycolysis, and found that inhibiting oxidized ATM using KU60019, or knockdown of ATM in Hs578T and BT549, decreased glucose consumption of CSC spheres under hypoxia (Fig. 3c). Correspondingly, the significant reduction of pyruvate products was also detected in oxidized ATM-inactivated or ATM-silenced TNBC-CSCs (Fig. 3d). Interestingly, there was no significant change of lactate production (Fig. 3e). These data indicated an atypical Warburg effect of EMR, in which a high level of glycolysis was associated with a higher level of pyruvate rather than lactate in TNBC-CSCs.

To understand the effect of oxidized ATM on mitochondrial metabolism, we measured the representative intermediates in TCA cycle. We found that acetyl-CoA and citrate had a notable drop under inhibition of oxidized ATM or knockdown of ATM in mammospheres (Fig. 3f, g). However, there was mild decrease in succinate and fumarate, which were downstream of citrate in TCA cycle metabolites, when inhibiting oxidized ATM or silencing ATM in the mammospheres (Fig. 3h, i). To further understand the oxidized ATM-mediated metabolic characteristics in CSC, metabolic-tracing analysis with uniformly labeled ^13^C_6_-glucose was used. The distribution of isotopologs within the metabolites showed that there were a substantial m + 3-labeled pyruvate and citrate (containing two ^13^C atoms), but predominantly m + 0 succinate and fumarate (containing no or a few of ^13^C atoms) in hypoxic CSC (Fig. 3j). These data demonstrate that the energy flux from glycolysis mainly affluxes into mitochondria, and mitochondrial citrate mainly transports to the cytoplasm, rather than all of energy flux from citrate affluxes into downstream TCA cycle in TNBC-CSCs under hypoxia (Supplementary Fig. S2D).

Mitochondrial citrate transported into the cytoplasm and mainly metabolized into cytosolic acetyl-CoA by ACLY; inhibition of ACLY activity can disrupt the use of mitochondria-derived citrate, and mainly results in the decreased cytosolic acetyl-CoA and accumulation of cytosolic citrate and lactate^31^. Indeed, there was a significant increased lactate (Fig. 3k), mild increased products of succinate (Fig. 3l), and reduced cytosolic acetyl-CoA (Fig. 3m) in response to the administration of BMS303141 (a specific inhibitor of ACLY), suggesting that cytosolic acetyl-CoA is the major metabolite of mitochondria-derived citrates in hypoxic TNBC-CSCs. To exclude the potential negative impacts of chemical inhibitor KU60019 to cell viability, which may influence the metabolism of Hs578T and BT549-derived mammospheres, cell proliferation and cell death were checked. KU60019 treatment (5–10 μM) could halt proliferation of CSC, but almost had no significant effects on cell death (Supplementary Fig. S2E, F), indicating that inactivation of oxidized ATM, rather than its potential negative impacts of KU60019 to cell death, leads to reduced CSC formation and spheres sizes. Together, these data suggest that oxidized ATM induces a specific EMR, which possesses a high-level glycolysis and a relatively high activated mtOXPHOS and these glucose-derived carbon atoms are transported to the cytoplasm via citrate, which causes the accumulation of acetyl-CoA in hypoxic TNBC-CSCs.

### Oxidized ATM-mediated EMR fuels acetyl-CoA production

**Fig. 4:**
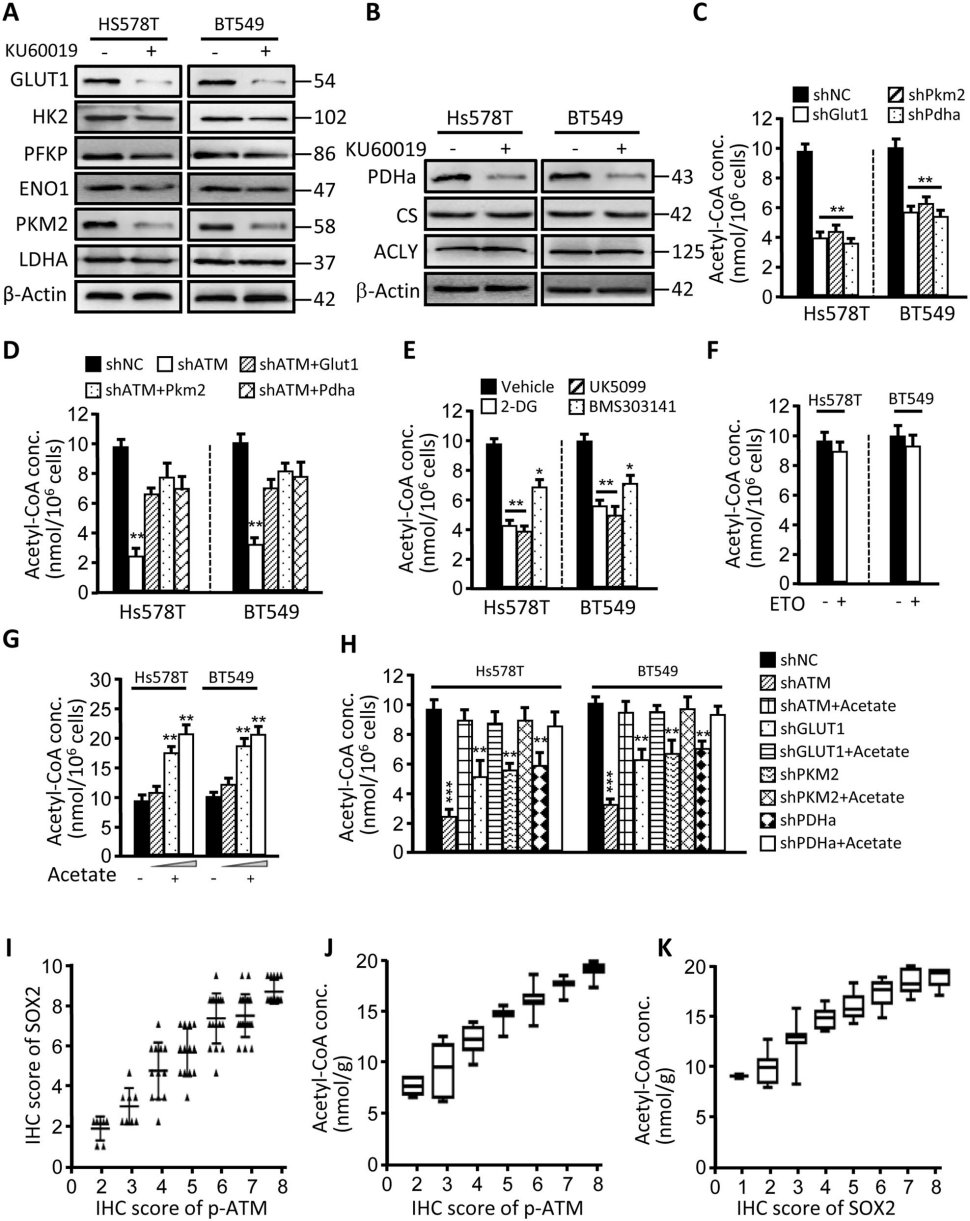
Oxidized ATM-mediated EMR fuels Ac-CoA production. **a**, **b** Western blotting show the glycolysis-related (**a**) and TCA-cycle-related (**b**) gene expressions in mammospheres derived from Hs578T and BT549 cells under treatment with or without KU60019 (10 µM) and from ATM-silenced cells in hypoxia. **c**–**h** Acetyl-CoA productions of the indicated mammospheres were determined. **c** Knockdown of GLUT1, PKM2, or PDHa and the control mammospheres. **d** Ectopic GLUT1, PKM2, or PDHa was transfected into hypoxic mammospheres with ATM knocked down. **e** Mammospheres treated with or without 2-deoxyglucose (2-DG, 5 mM), UK5099 (an inhibitor of the mitochondrial pyruvate carrier (MPC), 10 μM), or BMS303141 (an inhibitor of ACLY, 20 μM); **f** mammospheres treated with or without Etomoxir (ETO, an inhibitor of CPT1, 20 μM); **g** exogenous addition with or without acetate (1, 5, or 10 mM). **h** Exogenous addition with or without acetate (10 mM) in mammospheres under ATM, GLUT1, PKM2, or PDHa knocked down. **i**, **j** A positive correlation between p-ATM and SOX2 protein levels (**i**), p-ATM and production of acetyl-CoA (**j**), and SOX2 protein expression and acetyl-CoA (**k**) in 92 clinical breast cancer samples (*P < 0.05; **P < 0.01).

To uncover the potential genes regulated by oxidized ATM in inducing the atypical EMR of hypoxic TNBC-CSCs, we tested the glycolysis- and TCA-related gene expressions in oxidized ATM-activated and -inactivated mammospheres. Glycolysis-associated genes, GLUT1, PKM2, and TCA-related genes PDHa (encoding an enzyme catalyzing pyruvate into acetyl-CoA), were significantly down-regulated in mammosphere cells after impeding oxidized ATM activation or knockdown of ATM in hypoxia (Fig. 4a, b; Supplementary Fig. S3a, b), and were enhanced in hypoxic mammosphere cells compared with normoxic mammosphere cells (Supplementary Fig. S3C). Analyzed by bioinformatics, FOXP3 and C/EBPa were the potential transcription factors to GLUT1, PKM2, and PDHa (Supplementary Fig. S3D, E). Indeed, FOXP3 could transcriptionally regulate GLUT1, PKM2, and PDHa expressions by reporter assay and CHIP assay (Supplementary Fig. S3F, G). Knockdown of FOXP3 resulted in reduced GLUT1, PKM2, and PDHa protein levels in hypoxic Hs578T cells (Supplementary Fig. S3H, I). To understand whether oxidized ATM involved in up-regulating FOXP3, we reanalyzed our previous phosphoproteome data of hypoxic BT549; STAT5 is a target of activated ATM. Fortunately, FOXP3 was potentially regulated by STAT5 signaling^32^. As predicted, a high level of FOXP3 was detected in hypoxic CSCs, and decreased in KU60019-treated or ATM-silenced cells; STAT5-IN-1 (STAT5 inhibitor) treatment also mitigated FOXP3 expression of hypoxic CSCs (Supplementary Fig. S3J). These data demonstrate that oxidized ATM can up-regulate GLUT1, PKM2, and PDHa expressions via STAT5–FOXP3 signaling.

Next, we asked whether the EMR-associated gene alteration could affect acetyl-CoA production to fuel CSC enrichment under hypoxia. Knockdown of GLUT1, PKM2, or PDHa (Fig. S4A–C) in hypoxic Hs578T and BT549 decreased acetyl-CoA production (Fig. 4c) and mammosphere formation (Supplementary Fig. S4D). Furthermore, transfection of GLUT1, PKM2, or PDHa into ATM knockdown hypoxic BSCSs partly rescued acetyl-CoA production (Fig. 4d) and mammosphere formation (Supplementary Fig. S4E). Similarly, application of 2-DG, UK5099 (an inhibitor of the mitochondrial pyruvate carrier (MPC)), or BMS303141 (an inhibitor of ACLY) to hypoxic BT549 and Hs578T to interfere glycolysis, pyruvate import to mitochondria, or impeding cytosolic acetyl-CoA production caused a notable reduction of acetyl-CoA (Fig. 4E), which accordingly attenuated spheroid formations of CSC under hypoxia (Supplementary Fig. S4F). However, by administration of hypoxic CSC with Etomoxir (ETO, an inhibitor of CPT1) to intervene potential acetyl-CoA synthesis from fatty acid catabolism, there were no significant changes of acetyl-CoA concentrations in Hs578T and BT549 mammospheres (Fig. 4f) and corresponding alteration of sphere number and size (data not shown). The above data indicate that oxidized ATM enhanced the enrichment of BCSCs via regulating the accumulation of acetyl-CoA, and oxidized ATM increased the production of acetyl-CoA, which mainly came from glucose, through enhancing the expression of GLUT1, PKM2, and PDHa. Adding acetate (a precursor of acetyl-CoA)^33^ to the medium could increase the acetyl-CoA concentrations of hypoxic Hs578T and BT549 mammospheres (Fig. 4g) and promote the CSC formation in a dose-dependent manner (Supplementary Fig. S4G). Furthermore, exogenous acetate could partly restore acetyl-CoA production of mammosphere cells, which was compromised by knockdown of ATM or silencing EMR-regulated genes (e.g. GLUT1, PKM2, and PDHa) expressions (Fig. 4h). Correspondingly, exogenous acetate partly rescued the TNBC-CSC formation, which was blunted by KU60019, or knockdown of GLUT1, PKM2, and PDHa gene expressions (Supplementary Fig. S4H), indicating that acetyl-CoA associated with oxidized ATM-mediated EMR promotes TNBC-CSC sphere formation. To support these findings, we tried to measure the amounts of acetyl-CoA in tumor tissues and detect the levels of p-ATM and SOX2. A positive correlations among the levels of p-ATM, SOX2, and acetyl-CoA products was found in 92 clinical breast tumors (Fig. 4i–k). Together, these data unravel that oxidized ATM-mediated EMR facilitates glycolytic flux to pyruvate and citrate, thus fueling acetyl-CoA production in the cytoplasm to profit mammosphere formation.

### Acetyl-CoA regulates acetylation of histone H4 and CSC-associated gene expressions

**Fig. 5:**
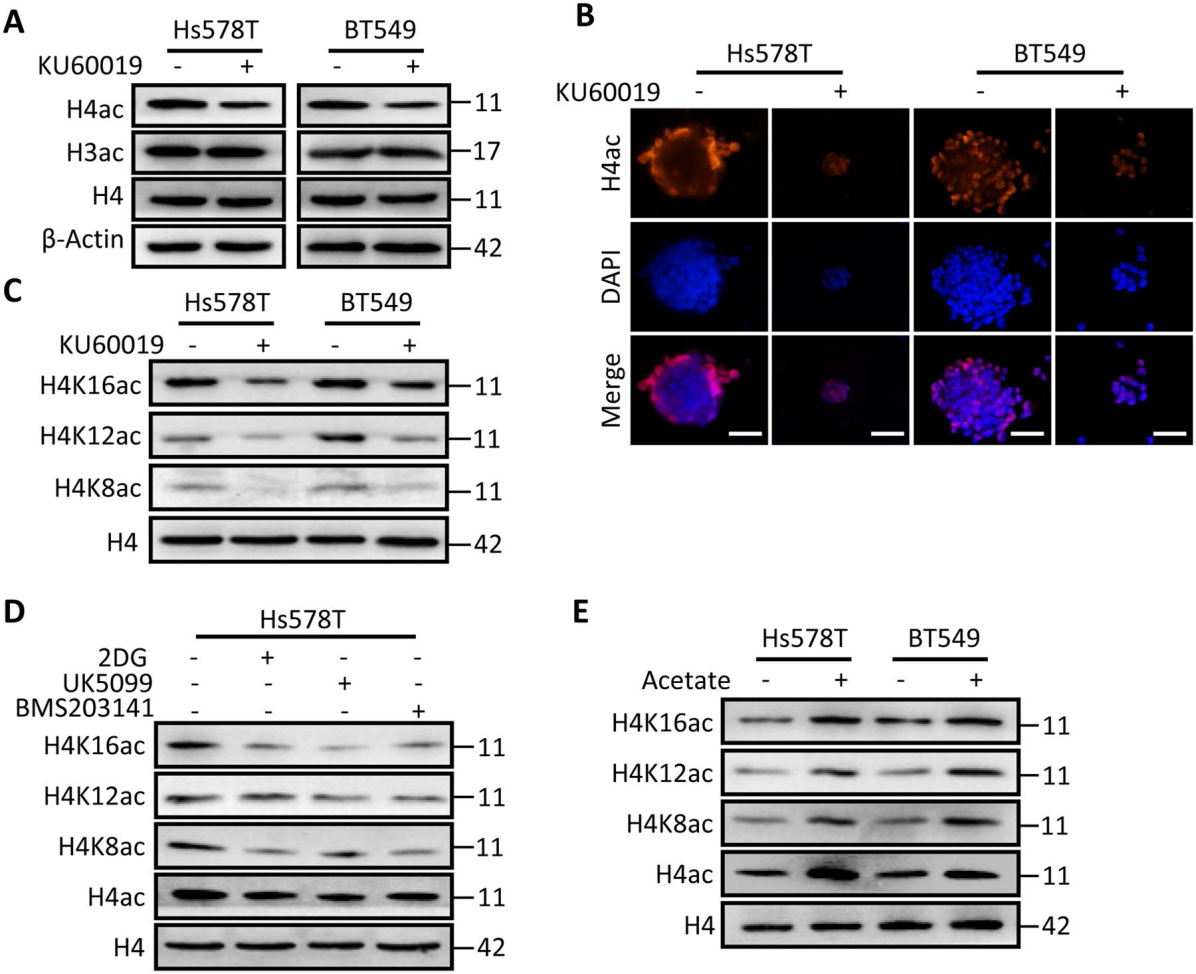
Acetyl-CoA regulates acetylation of H4 histones and CSC-associated gene expressions. **a** Protein levels of acetylated-lysine histone H3 and H4 were determined by immunoblotting in cell lysates from Hs578T and BT549 spheres treated with or without KU60019 (10 μM) under hypoxia. β-Actin is the loading control. **b** Immunofluorescence staining to check acetylated-H4ac levels in Hs578T and BT549 mammospheres treated with or without KU60019 (scale bar, 100 µm) under hypoxia. **c**–**e** The status of H4 acetylation was determined in hypoxic Hs578T and/or BT549 mammospheres suffered to different treatments. **c** Spheres treated with KU60019 (10 μM); **d** those treated with 2-DG (5 mM), UK5099 (15 μM), and BMS303141 (20 μM), respectively; **e** those treated with acetate (10 mM). Total H4 is the loading control.

To ask whether oxidized ATM-mediated acetyl-CoA accumulation contributes to histone acetylation, the acetylation of H3 and H4 was checked. Interestingly, the acetylation of total histone H4, rather than H3, was decreased in oxidized ATM-inactivated mammosphere cells detected by western blotting (Fig. 5a) and immunofluorescence staining (Fig. 5b). To identify which lysine residues at histone H4 were acetylated, we performed western blotting using antibodies specifically against histone H4 acetyl lysine residues, and found that acetylation of H4K8ac, H4K12ac, and H4K16ac was obviously decreased (Fig. 5c), suggesting that oxidized ATM maintains the acetylation levels of histone H4.

Our above data showed that impeding glycolysis inhibiting pyruvate into mitochondria or disrupting citrate into cytosolic acetyl-CoA caused a marked drop in cellular acetyl-CoA levels in mammosphere cells under hypoxia (Fig. 4e). We next wondered whether interfering EMR or citrate cleavage in the cytoplasm could alter histone H4 acetylation. As expected, administering mammospheres with inhibitors (2-DG, UK5509, and BMS301341) mitigated the acetylation of H4K8ac, H4K12ac, and H4K16ac in hypoxic Hs578T (Fig. 5d) and BT549 (Supplementary Fig. S5A) mammospheres. In line with these findings, the mammosphere formation was notably decreased (Supplementary Fig. S4F), and CSC stemness-associated proteins were correspondingly reduced in the sphere cells treated with 2-DG, UK5099, and BMS301341, respectively (Supplementary Fig. S5B, C). Addition of acetate increased the acetylation levels of histone H4K8ac, H4K12ac, and H4K16ac (Fig. 5e), CSC spheres formation (Supplementary Fig. S4G), and the expressions of CSC-associated genes (Supplementary Fig. S5D). These data confirm that accumulation of glycolysis-derived acetyl-CoA promotes histone H4 acetylation and fuels CSC-associated gene expressions.

### Oxidized ATM promotes tumor formation of CSCs in mice xenografts

**Fig. 6:**
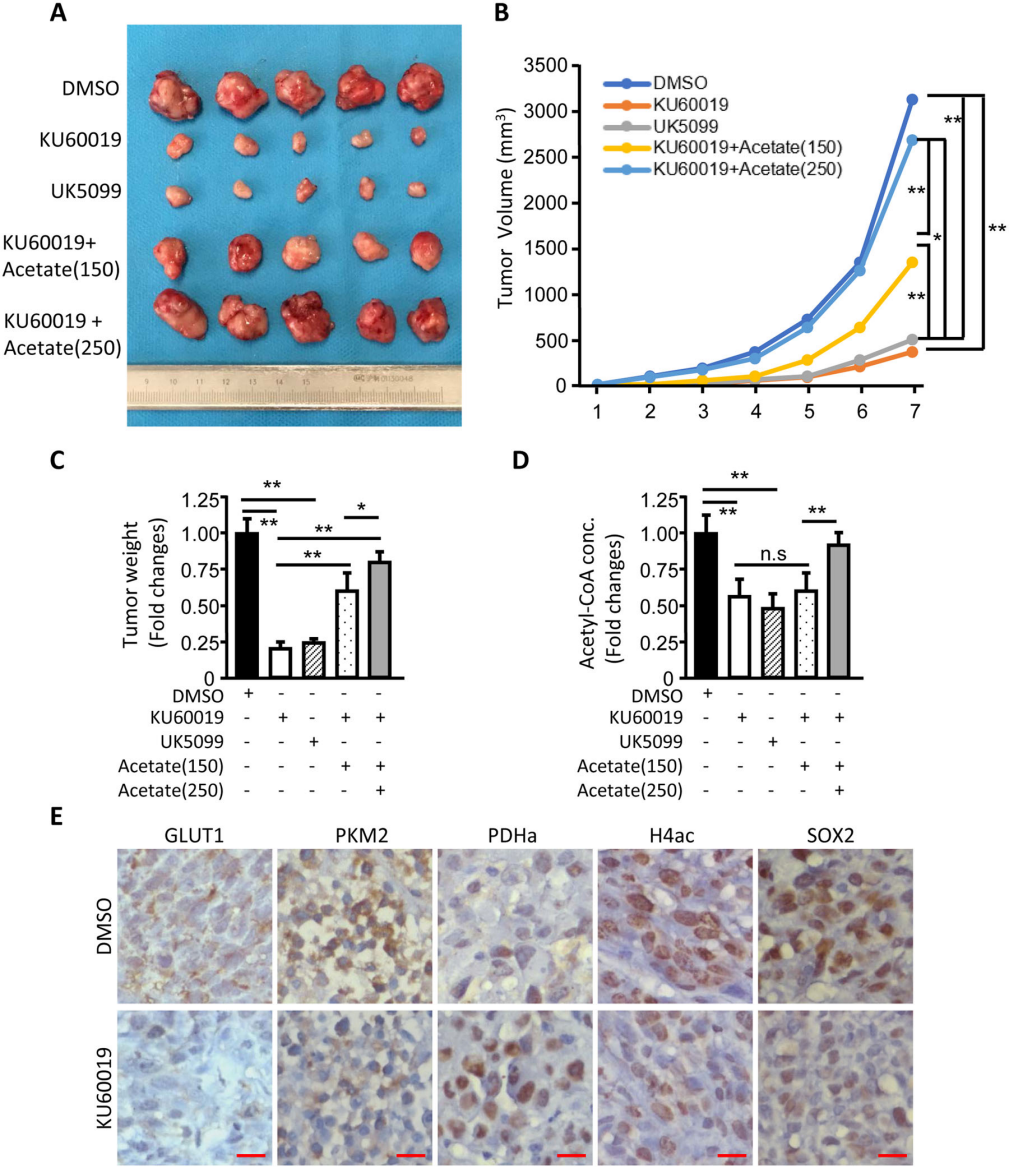
Oxidized ATM-mediated EMR promotes tumor formation of CSCs in mice xenografts. **a** Photos of the xenograft tumors dissected from the nude mice at the seventh week after being subcutaneously injected with BT549 mammosphere cells. When tumor volume reaches around 50 mm^3^, mice were randomized into five groups and administered with KU60019 (50 mg/kg, three times a week), or UK5099 (10 μg/kg, once every 2 days), acetate (150 mg/kg, three times a week), and acetate (250 mg/kg, three times a week) alone or in combination, via intraperitoneal (i.p.) injection. **b** Tumor growth curve (*P < 0.05, **P < 0.01). **c** The average tumor weight of each group. **d** Acetyl-CoA productions of xenograft tumors. **e** GLUT1, PKM2, PDHa, H4ac, and SOX2 levels were determined in tumor tissues by IHC analyses.

To investigate the role of oxidized ATM on tumorigenesis in vivo, we established TNBC-CSC xenograft models. As shown in Fig. 6a–c, oxidized ATM promoted CSCs to initiate tumor formation and growth in nude mice; administering nude mice with KU60019 as well as UK5099 delayed tumorigenic ability and suppressed tumor growth; exogenous acetate had an antagonistic ability to KU60019 and UK5099 to fuel the initiation of tumor formation and tumor growth of tumor-burden mice treated in a dose-dependent manner (Fig. 6a–c). Consistently, the acetyl-CoA levels in the relevant xenograft tumors had similar changes (Fig. 6d). These findings indicate that the activation of oxidized ATM and the corresponding acetyl-CoA accumulation promote tumor formation and growth in vivo.

To understand the causal correlation among oxidized ATM activation, EMR, and CSC formation in vivo, IHC analyses were performed to test GLUT1, PKM2 and PDHa, H4ac and SOX2 levels in these xenograft tumors. As shown in Fig. 6e, KU60019-mediated oxidized ATM inactivation correlated with the decreased GLUT1, PKM2 and PDHa, H4ac and SOX2 levels in xenograft tumors in comparison with the oxidized ATM-activated tumors, indicating that oxidized ATM facilitates tumorigenesis of TNBC-CSC and tumor growth via EMR-related acetyl-CoA accumulation.

## Discussion

TNBC is a breast cancer subtype with a high risk of recurrence and metastasis. Intratumoral hypoxia is one crucial hallmark of solid tumor microenvironment which may strongly promote an aggressive tumor phenotype resulting in therapeutic resistance and poor patient survival^15,16^. Indeed, recent studies unravel that TNBC patients who accepted traditional therapeutic strategies represent failure to treatment due to TNBC-CSC augment through the hypoxia pathway^4,5^. However, the specific mechanism of hypoxia-induced CSC enrichment is unclear. Here, we provide evidence to support the functional roles of hypoxia-induced DNA damage-independent ATM activation (oxidized ATM) in stemness maintenance of TNBC-CSCs. In this study, oxidized ATM was detected in normoxic TNBC-CSCs, and more significantly enhanced in the hypoxic TNBC-CSCs. We found that oxidized ATM induces a specific EMR, which facilitates glucose uptake and glycolytic flux to pyruvate and citrate, thus fueling acetyl-CoA production in the cytoplasm to profit mammosphere formation. Thus, oxidized ATM-mediated acetyl-CoA production maintains histone H4 acetylation level that in turn increases the CSC-associated gene expressions and promotes TNBC-CSC formation and stemness maintenance.

Low oxygen tensions can straigh away affect the pluripotency or differentiation of stem cells. It was found that hypoxic culture prevents differentiation of hES cells^17^ and promotes an undifferentiated and multipotent status in MSC^18^. Hypoxia was also found to facilitate CSC enrichment by activiating HIF1α/2α and AKT/mTOR/β-catenin pathways^20^. In our study, we found that hypoxia-induced oxidized ATM could enhance CSC formation and stemness maintenance of hypoxic TNBC cells. Inhibition of oxidized ATM kinase by the specific inhibitor KU60019 or knockdown of ATM gene significantly reduced mammosphere formation of hypoxic TNBC cells. Actually, the cytoplasmic ATM could be activated by hypoxia^25^ in DSB-dependent manner, and involves in intracellular redox homeostasis, metabolism reprogramming^25,26^, and self-renewal capacity of embryonic stem cells^27^. These data demonstrate that the oxidized ATM plays a pivotal role to ES and CSC formation and stemness maintenance.

Several studies suggest that CSCs have a changed energy metabolism pattern. The CSCs derived from lung cancer, ovarian cancer, glioblastoma, osteosarcoma, breast cancer, and colon cancer are more glycolytic than their differentiated cancer cells^34–37^. The latest study reveals that the increased activation of mitochondrial oxidative phosphorylation in TNBC, inhibition of mitochondrial function by metformin, or oligomycin A reduced mammosphere formation^5^. However, evidence for the metabolic pattern under hypoxic conditions is inconclusive. In this study, we found that hypoxic TNBC-CSCs displayed high glycolytic capacity and relatively low mitochondrial respiration that may be closely associated with oxidized ATM activation under hypoxia. Similarly, other reports have also indicated that oxidized ATM is involved in EMR. For example, hypoxia-activated TRAF6 constitutes the ATM-γH2AX-HIF1α pathway and resulted in more glucose uptake and glycolysis in hypoxic tumors^25^. Interestingly, we found that oxidized ATM-mediated an increased glucose uptake and glycolytic metabolite pyruvate rather than lactate in hypoxic TNBC-CSCs. The increased glycolytic flux mainly affluxes into mitochondrial acetyl-CoA and citrate. More importantly, the mitochondrail citrate could be exported into the cytosol, rather than fed into the TCA cycle, where it can be cleaved by ACLY to produce cytosolic acetyl-CoA. Thus, the oxidized ATM sustains an atypical Warburg effect of EMR in TNBC-CSCs. Similar phenomenon has also been observed in hESC, mESC, and NK cells^11,31^. The adaptive metabolic plasticity mightallow CSCs, for example, CD110^+^ colorectal cancer tumor-initiating cells (TICs), brain tumor-initiating cells, and resistant CSC clones, to survive in changeable, especially in hostile metastatic environments or unfavorable circumstances^38^. These findings imply that there is inherent connection between oxidized ATM-induced variety of metabolic pattern and CSC formation.

Metabolic reprogramming is an early contributor to the orchestrated departure from or reacquisition of stemness^39^. Some intermediates regulate epigenetic and transcriptional changes associated with maintenance of SC’s stemness and self-renewal, including acetyl-CoA^11^, lactate^40^, α-KG^41^, and NAD^+^^12^. In the present work, we found that oxidized ATM-mediated EMR-related acetyl-CoA accumulation plays an essential role in TNBC-CSCs formation and stemness maintenance. Oxidized ATM in TNBC-CSC facilitates the expressions of GLUT1, HK2, PKM2, and PDHa, leading to more glucose upatake, glycolysis, and citrate production, which result in the accumulation of cytosolic acetyl-CoA. Inhibition of oxidized ATM, glycolysis, the transport of pyruvate into mitochondria, or impeding ACLY activity significantly reduced the acetyl-CoA productions and CSC maintenance, supporting the concept that TNBC-CSC formation and stemness maintenance are dependent on oxidized ATM-induced acetyl-CoA accumulation.

Histone acetylation is critical in maintaining an open-chromatin structure, and acetyl-CoA is a major donor to histone acetylation^8,9^. Under hypoxia conditions, we found that mammosphere cells derived from DNA damage-independent ATM-inactivated TNBC, the acetylation of histone H4 was decreased. Administration of TNBC-derived CSCs with 2-DG, UK5509, and BMS301341 resulted in reduced acetyl-CoA and suppressed the acetylation of histone H4. In contrast, treatment of CSC with EX527 or joint administration with acetate recuperated the H4 histone acetylation of CSC, and promoted CSC-associated gene expression and CSC formation. Our study uncovers that oxidized ATM governs acetyl-CoA accumulation, promotes hypoxic TNBC-CSC formation and stemness maintenance.

**Fig. 7:**
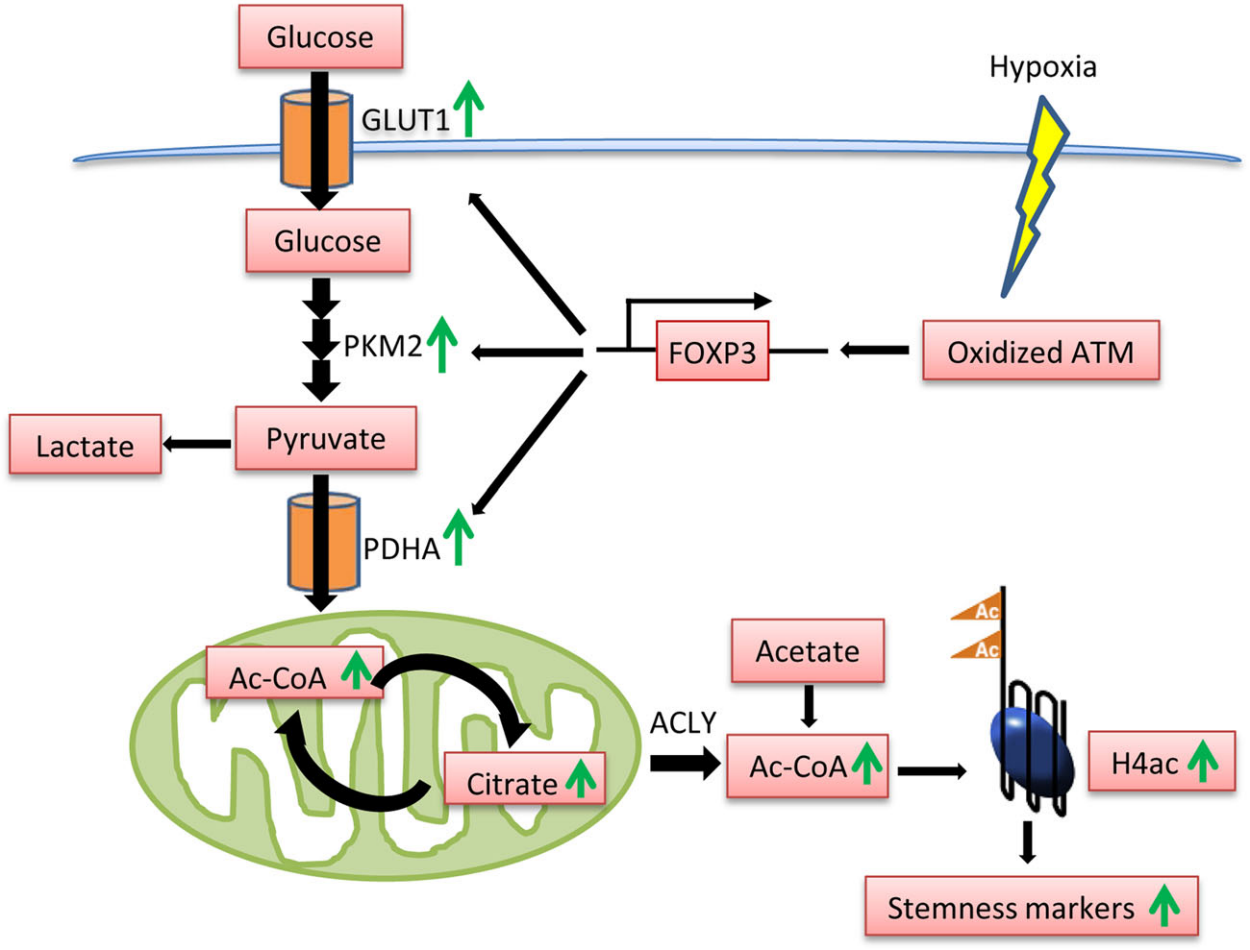
The schematic diagram of oxidized ATM in mediation of EMR, acetyl-CoA accumulation, and BCSCs formation. A schematic diagram depicts the functional roles of oxidized ATM in regulating atypical EMR, cytoplasmic acetyl-CoA accumulation involved in TNBC-CSC formation and stemness maintenance.

In summary, this work discovers a novel function of oxidized ATM in TNBC-CSC formation and stemness maintenance. The oxidized ATM-induced atypical EMR leads to intracellular acetyl-CoA accumulation that, in turn, regulates histone H4 acetylation and CSC-associated gene expressions (Fig. 7). Taken together, the novel mechanism behind hypoxia-stimulating CSC formation and stemness maintenance may help us to develop new therapeutic strategies for TNBC treatment.

## Supplementary information

Figure S1

Figure S2

Figure S3

Figure S4

Figure S5

Supermentary Figure Legends
