# Supplementary figures and images for: Oxidized ATM promotes breast cancer stem cell enrichment through energy metabolism reprogram-mediated acetyl-CoA accumulation
Source: Cell Death Dis. 2020 Jul 3;11(7):508. doi: 10.1038/s41419-020-2714-7 (PMC7343870; doi:10.1038/s41419-020-2714-7)

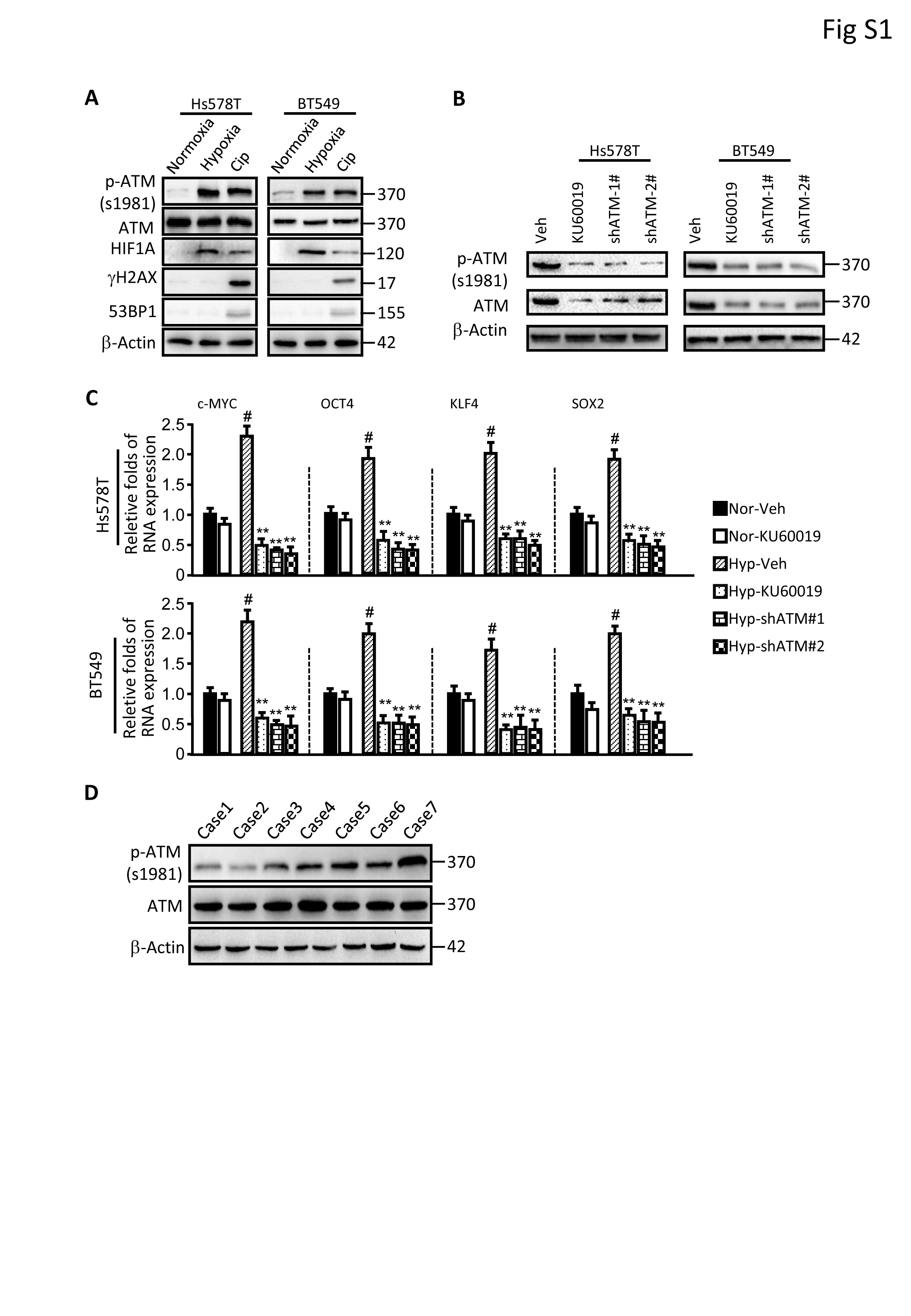

Supplement: Supplementary file 1 — Figure S1 [file 41419_2020_2714_MOESM1_ESM.tif]

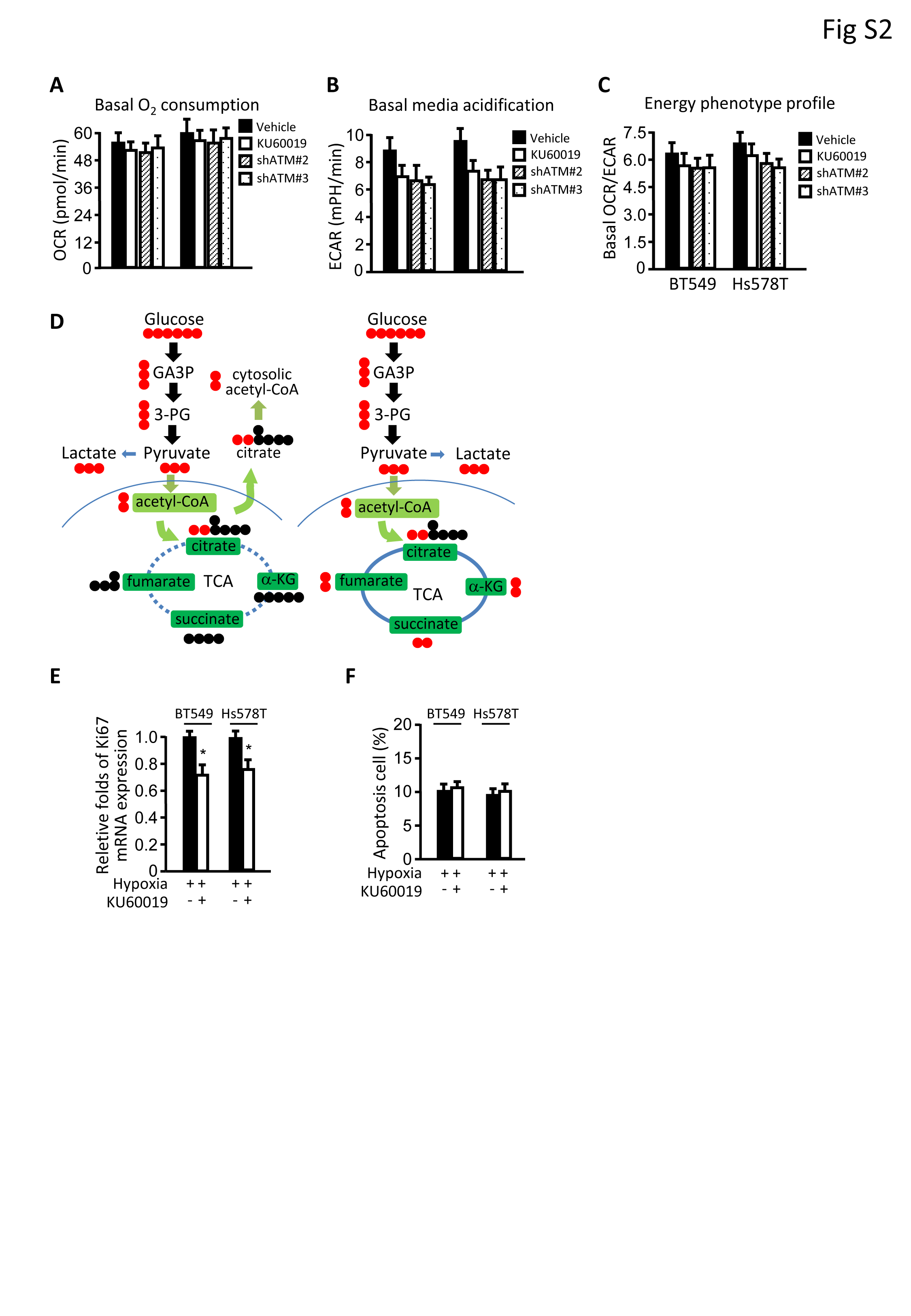

Supplement: Supplementary file 2 — Figure S2 [file 41419_2020_2714_MOESM2_ESM.tif]

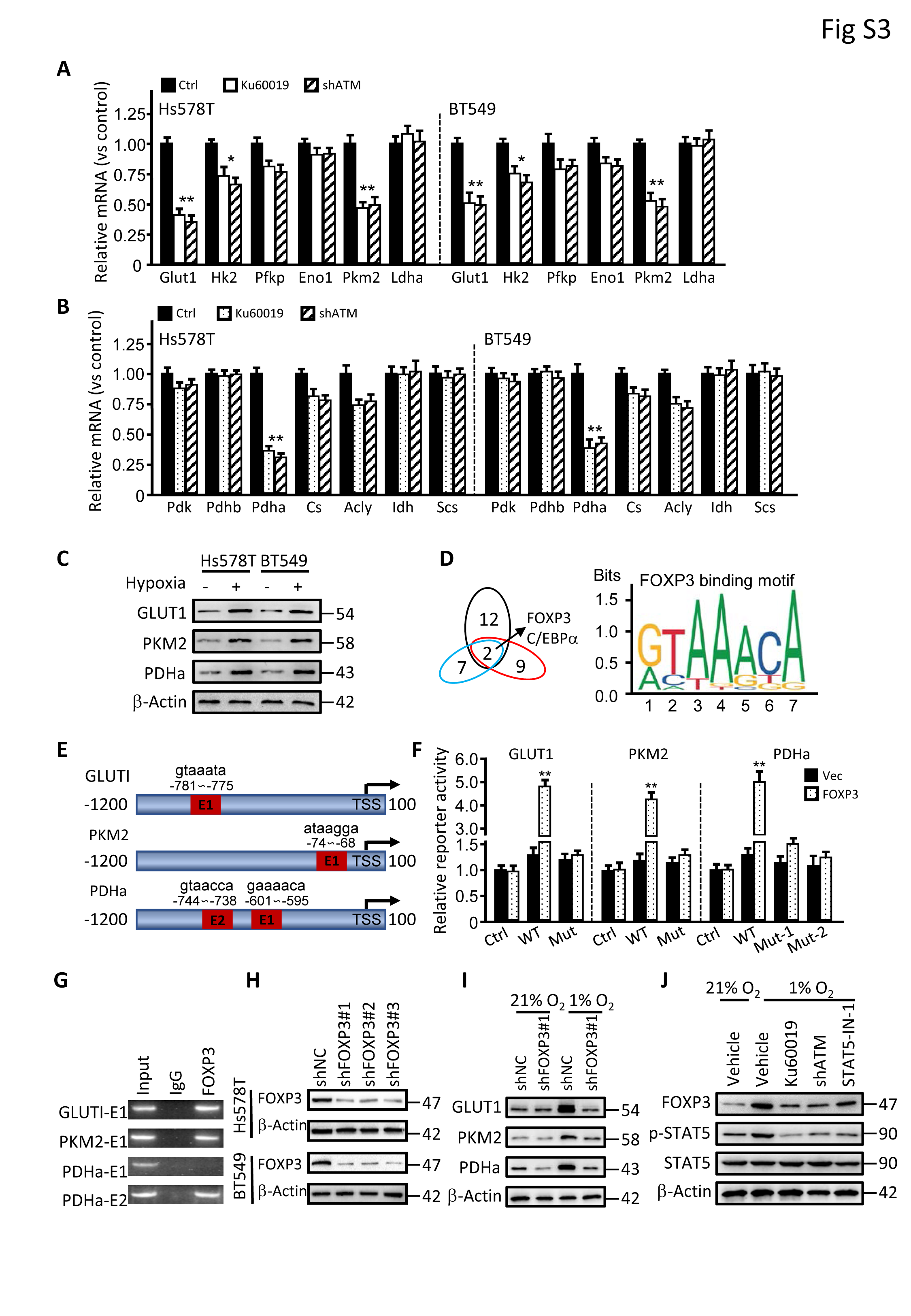

Supplement: Supplementary file 3 — Figure S3 [file 41419_2020_2714_MOESM3_ESM.tif]

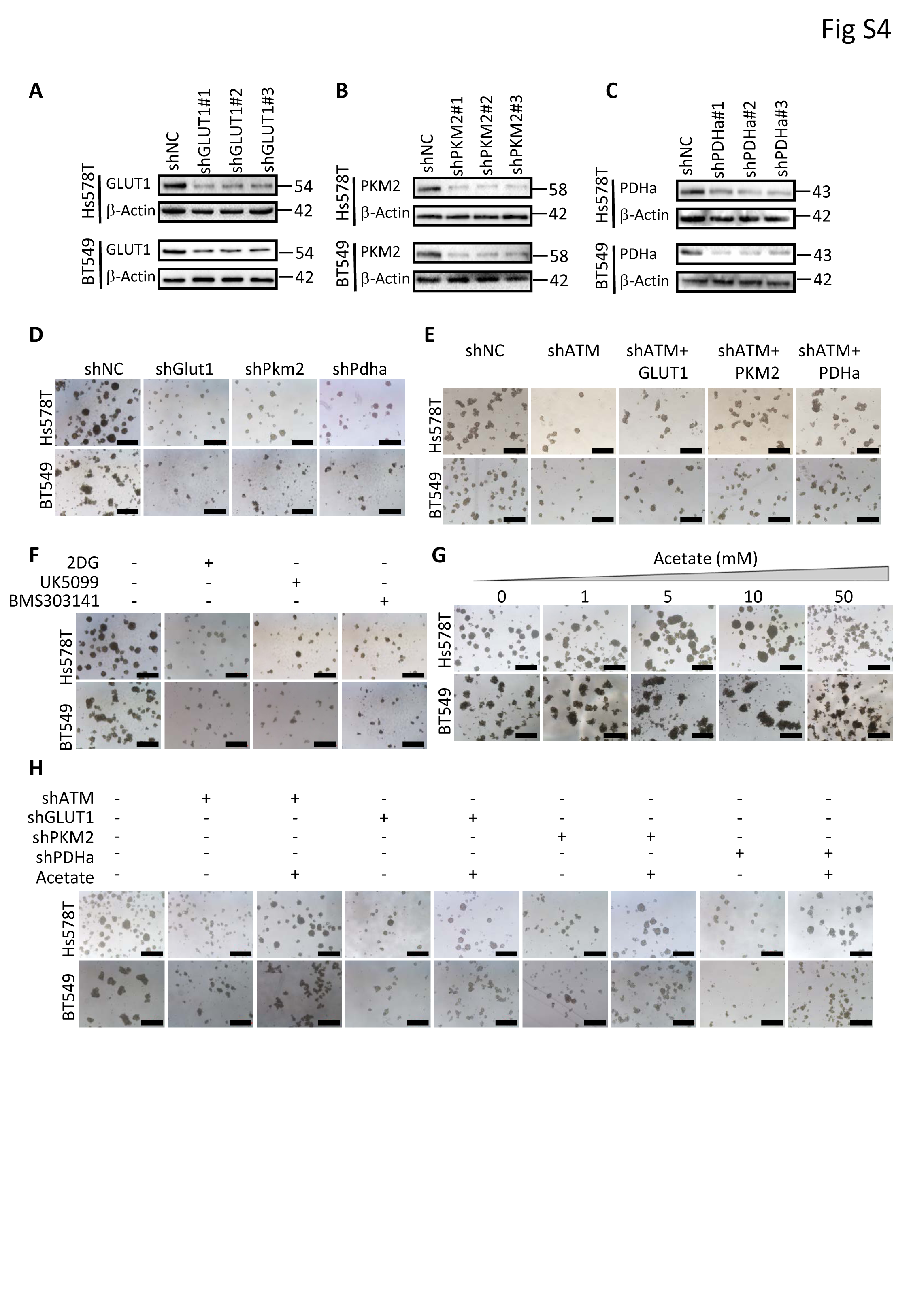

Supplement: Supplementary file 4 — Figure S4 [file 41419_2020_2714_MOESM4_ESM.tif]

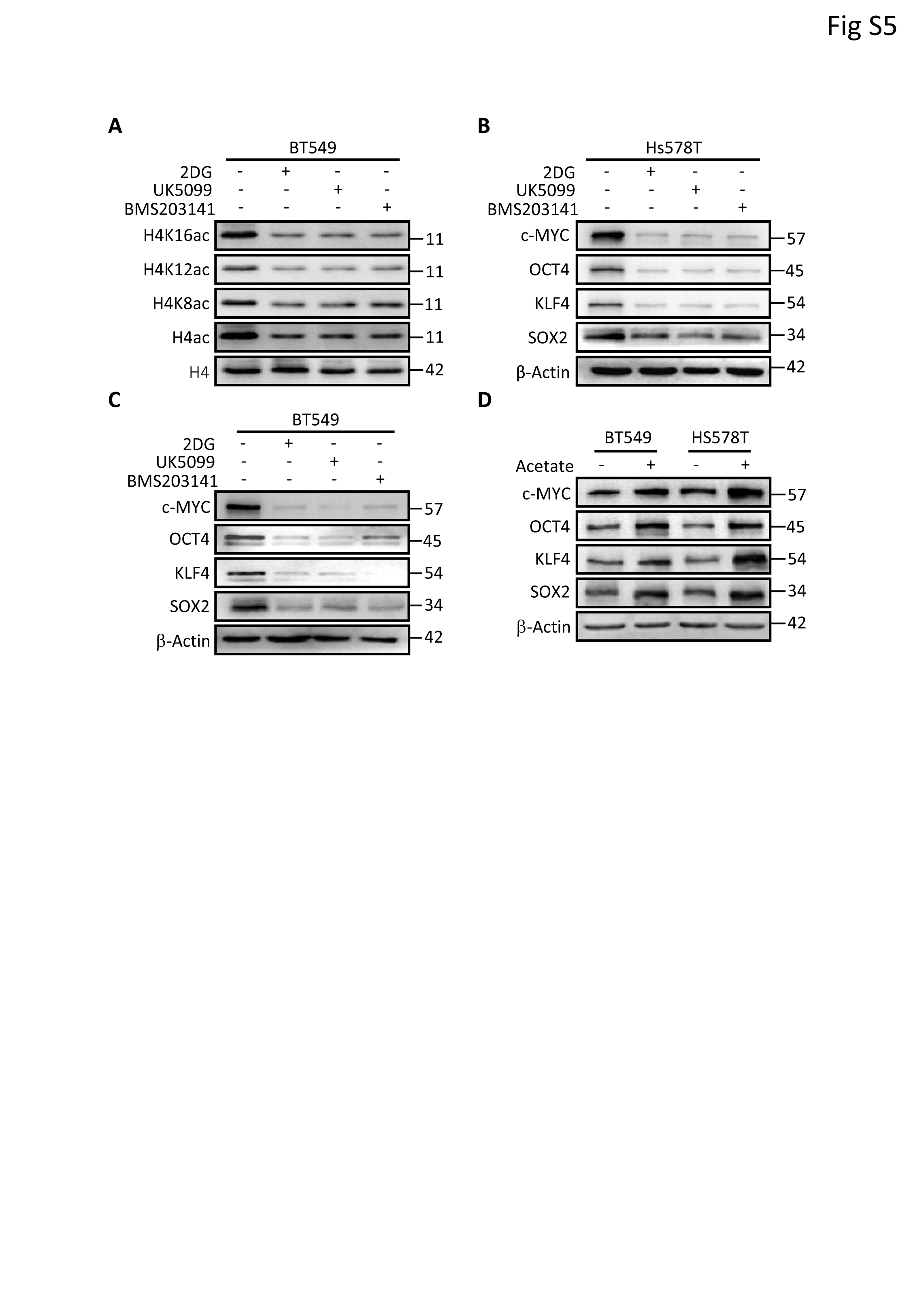

Supplement: Supplementary file 5 — Figure S5 [file 41419_2020_2714_MOESM5_ESM.tif]
