# Supplementary material for: Oxidized ATM promotes breast cancer stem cell enrichment through energy metabolism reprogram-mediated acetyl-CoA accumulation
Source: Cell Death Dis. 2020 Jul 3;11(7):508. doi: 10.1038/s41419-020-2714-7 (PMC7343870; doi:10.1038/s41419-020-2714-7)
Supplement: Supplementary file 6 — Supermentary Figure Legends [file 41419_2020_2714_MOESM6_ESM.docx]

**Supermentary Figure Legends**

**Figure S1. Related to Figure 2: Oxidized ATM activity and CSC-associated genes expressions**

(A). Western blotting to check the oxidized ATM activity (p-ATM (s1981)), two of known DNA damage protein markers (γH2AX and 53BP1) in response to hypoxia, cisplatin (10 μg/ml) treatment worked as positive control to DNA damage. (B). The efficiencies of ATM silence or inhibition in the indicated cells determined by western blotting. (C). qRT-PCR to determine the indicated CSC-associated gene expressions in mammospheres derived from Hs578T and BT549 under the indicated treatment or from ATM-knocked down Hs578T and BT549 cells. (D). Western blotting to check oxidized ATM, total ATM expressions in 7 tumors randomly selected from breast cancer patients.

**Figure S2. Related to Figure 3: ECAR and OCR detection, and metabolism pattern in mammospheres**

(A-C). Mitochondrial activity (OCR) and glycolytic (ECAR) of the indicated mammospheres were analyzed by Glyco Stress Test and Mito Stress Test using a Seahorse XF^e^96 extracellular flux analyzer, respectively. (A) the basal OCR, (B) the basal ECAR, (C) the cell energy phenotype profile (OCR/ECAR) were shown as bar diagrams. (D). The expected ^13^C labeling patterns of glycolysis and TCA-cycle metabolites with ^13^C_6_-glucose as tracer. (E, F). KU60019 (10 μM) was used to treat mammospheres, the effects of KU60019 on spheres survival and cell death were tested by Ki67 mRNA expression and apoptosis cells.

**Figure S3. Related to Figure 4: glycolysis related genes regulated by DSBsi ATM**

(A, B). qRT-PCR show the glycolysis-related (A) and TCA-cycle related (B) gene expressions in mammospheres derived from Hs578T and BT549 cells under treatment with or without KU60019 (10 µM) and from ATM-silenced cells in hypoxia. (C). The expression of GLUT1, PKM2 and PDHa were tested by western blotting. (D). Bioinformatics analysis of potential transcriptional factors (left panel) for Glut1 (black cycle), Pkm2 (red cycle) and Pdha (blue cycle), two of common transcriptional factors, FOXP3 and C/EBP, were identified. FOXP3 binding motif in the Glut1, Pkm2 and Pdha gene promoter (right panel) are shown. (E). Potential FOXP3 binding motif and site at Glut1, Pkm2 and Pdha gene promoter. (F, G). Transcriptional activity of Glut1, Pkm2 and Pdha gene regulated by FOXP3 evaluated using luciferase assay (F) and CHIP assay (G). Mut-1 or Mut-2 represented the mutant constructor of the E1 or E2 site in Pdha promoter, respectively. (H). The efficiencies of FOXP3 silence in the indicated cells determined by western blotting. (I). FOXP3 regulated GLUT1, PKM2 and PDHa protein levels in hypoxia and normoxia condition were determined by western blotting assay. (J). FOXP3 expression potentially regulated by STAT5 (STAT5-IN-1, a STAT5 inhibitor, 25 μM) was assessed using western blotting.

**Figure S4. Related to Figure 4: DSBsi ATM-mediated acetyl-CoA production promotes mammospheres formation**

(A). The efficiencies of Glut1 silence in the indicated cells determined by western blotting. (B). The efficiencies of PKM2 silence in the indicated cells determined by western blotting. (C). The efficiencies of PDHa silence in the indicated cells determined by western blotting. (D). Reprehensive images of mammospheres from GLUT1-, PKM2- or PDHa-knocked down Hs578T and BT549, and their control cells. (E). Ectopic GLUT1, PKM2 or PDHa was transfected into hypoxic mammospheres with ATM-knocked down, and representative images of spheres were shown. (F). Representative images of spheres from Hs578T, BT549 treated with or without 2-DG (5 mM), UK-5099 (15 µM) or BMS303141 (20 μM), respectively. (G). Different dose of acetate were added to medium, the sphere formations of Hs578T and BT549 cells were checked under hypoxia. (H). Representative images of spheres from hypoxic Hs578T and BT549 with knocked down ATM, GLUT1, PKM2, PDHa alone or combined with addition of acetate (10 mM). Scale bar, 500 µm in pictures.

**Figure S5. Related to Figure 5: Acetyl-CoA regulates acetylation of H4 histones and CSC-associated gene expressions**

(A). Immunoblots of acetylated H4 proteins in BT549 mammospheres treated with 2-DG (5 mM), UK-5099 (15 μM) and BMS303141 (20 μM) under hypoxia condition, respectively. (B-C). The stemness markers in hypoxic Hs578T (B) and BT549 (C) spheres were measured by western blotting analysis. Spheres were treated with 2-DG (5 mM), UK-5099 (15 μM) and BMS303141 (20 μM), respectively. (D). The stemness markers in spheres formed from hypoxic Hs578T and BT549 treated with or without acetate (10 mM).
